# Controlled human exposures: a review and comparison of the health effects of diesel exhaust and wood smoke

**DOI:** 10.1186/s12989-024-00603-8

**Published:** 2024-10-23

**Authors:** Erin Long, Christopher F. Rider, Christopher Carlsten

**Affiliations:** 1 Faculty of Medicine, University of British Columbia, 317 – 2194 Health Sciences Mall, Vancouver, BC V6T 1Z3 Canada; 2 Department of Medicine, Division of Respiratory Medicine, University of British Columbia, 2775 Laurel Street 7th Floor, Vancouver, BC V5Z 1M9 Canada

**Keywords:** Diesel exhaust, Wood smoke, Air pollution, Controlled human exposures, Traffic-related air pollution, Biomass burning

## Abstract

One of the most pressing issues in global health is air pollution. Emissions from traffic-related air pollution and biomass burning are two of the most common sources of air pollution. Diesel exhaust (DE) and wood smoke (WS) have been used as models of these pollutant sources in controlled human exposure (CHE) experiments. The aim of this review was to compare the health effects of DE and WS using results obtained from CHE studies. A total of 119 CHE-DE publications and 25 CHE-WS publications were identified for review. CHE studies of DE generally involved shorter exposure durations and lower particulate matter concentrations, and demonstrated more potent dysfunctional outcomes than CHE studies of WS. In the airways, DE induces neutrophilic inflammation and increases airway hyperresponsiveness, but the effects of WS are unclear. There is strong evidence that DE provokes systemic oxidative stress and inflammation, but less evidence exists for WS. Exposure to DE was more prothrombotic than WS. DE generally increased cardiovascular dysfunction, but limited evidence is available for WS. Substantial heterogeneity in experimental methodology limited the comparison between studies. In many areas, outcomes of WS exposures tended to trend in similar directions to those of DE, suggesting that the effects of DE exposure may be useful for inferring possible responses to WS. However, several gaps in the literature were identified, predominantly pertaining to elucidating the effects of WS exposure. Future studies should strongly consider performing head-to-head comparisons between DE and WS using a CHE design to determine the differential effects of these exposures.

**Supplementary Information:**

The online version contains supplementary material available at 10.1186/s12989-024-00603-8.

## Background

Air pollution is one of the major threats to human health in the modern era [1], with approximately half the global population exposed to increasing levels of air pollution [2] and 94% of the world’s population exposed to particulate matter (PM) above the limits recommended by the World Health Organization [3]. It is estimated that more than six million deaths annually are attributable to air pollution [4]. Two of the most common sources of air pollution worldwide are traffic-related air pollution (TRAP) and biomass burning. TRAP has been associated with cardiovascular disease [5–8] and respiratory illness [9–13], among many other health consequences [14, 15]. Diesel exhaust (DE) engines in trucks, private motor vehicles, and trains are major contributors to TRAP, given their widespread use and popularity [16]. Biomass (i.e. organic matter from plants and animals, such as wood or manure) emissions, commonly derived from the burning of wood for residential heat or cooking [17, 18], as well as from wildfire smoke [19], have similarly been associated with a plethora of adverse health outcomes [17, 20–23].

Previously, we reviewed the health effects of DE based on evidence from controlled human exposure (CHE) studies to DE (CHE-DE) [24] as well as from CHE studies to wood smoke (WS) (CHE-WS) [25] as models for exposure to TRAP and biomass burning or wildfires, respectively. Controlled human exposures (CHE) are an experimental methodology that involves exposing participants to a known target concentration of a pollutant to evaluate the effects of the pollutant on various health outcomes. The advantage of this approach is the degree of control that can be achieved across multiple variables, allowing for stronger mechanistic insights to be made. CHE studies typically employ a crossover design in which all participants are exposed to all experimental and control arms, usually in a blinded fashion and randomized order, with a pre-determined washout period separating each exposure. This design enables each participant to act as their own control, greatly decreasing the impact of confounders, while also allowing for analysis of diverse variables (age, sex, genotype, etc.) that may modify response to the exposure in question. Virtually all CHE-DE and CHE-WS studies include a filtered air (FA) exposure as a control, to account for various extraneous or spurious factors, and subsequently better delineate the effect of the pollutant on the outcome examined.

While PM contributes to the overall makeup of both DE and WS, the physiochemical properties of PM vary, as do the non-PM fractions of each pollutant [25–29]. WS is comprised of three main classes of combustion particles: organic carbon, soot, and inorganic ash, with their relative contributions varying based on emission source (wildfire, residential wood stove etc.) and combustion conditions [25, 30]. The non-PM portion of WS differs by source and combustion condition as well, but major constituents typically include carbon monoxide (CO), carbon dioxide (CO_2_), and volatile organic compounds [27, 31–34]. In contrast, nitrogen oxides (NO_x_) are a main contributor to the non-PM fraction of DE, in addition to CO and hydrocarbons [34, 35]. Given the differing composition of DE and WS, a comparison of the resultant health effects would help inform how this translates to any meaningful clinical impact, tradeoffs in control measures, and associated policy. Indeed, there is literature that indicates different constituents of PM exert varying health effects [36–38]. In vivo studies have also demonstrated evidence of differential effects between WS and DE [34, 39–42]. Notably, while epidemiological evidence supports associations between non-PM fractions of air pollution and adverse health outcomes [43–45], studies examining the effects of WS and DE typically utilize PM as the primary benchmark for quantifying exposure. As such, this review will predominantly focus on the PM fractions of these exposures.

Should the health effects of biomass emissions be shown to be similar to those of DE, this could enable the propagation of policies developed during the extensive study of TRAP to the more novel context of WS exposure. Such improved knowledge could be translated accordingly into improved air quality standards, cost‒benefit analyses for policy and funding changes, air quality health index (AQHI) development, and ultimately medications to mitigate associated health problems. As such, in this review, we compare and contrast the health effects of WS and DE, as derived from CHE studies. We note that unless otherwise clarified, studies may be assumed to have utilized appropriate control exposures or groups.

## Methods

The Pubmed and Web of Science databases were searched for English-language articles about CHE to DE or WS. To search for CHE studies, queries included “controlled human exposure”, “human exposure”, “human” AND “exposure”. These search terms were used in combination with the following: “wildfire”, “woodsmoke”, “wood” AND “smoke”, and “diesel exhaust”. To be eligible, participants must have been exposed via inhalation to a controlled quantity of either WS or DE. Articles up to September 2023 were included. Abstracts and academic theses were excluded, and letters about diesel exhaust were not reviewed, as these generally undergo less stringent peer review and contribute more limited data relative to full research publications. However, for WS, letters were included given the paucity of eligible paper publications. In total, 119 CHE-DE publications and 25 CHE-WS publications were identified for review.

## Results

### Pulmonary effects

Both TRAP and biomass smoke exposure are associated with a variety of pulmonary conditions and resultant detrimental effects. The development of chronic pulmonary disease has been independently connected to TRAP [46–48] and biomass smoke [49–52]. TRAP has also been linked to increased asthma exacerbations [53, 54] as well as irritative respiratory symptoms [55–57]. Wood smoke exposure has been associated with an increased risk of lung cancer [20, 58, 59] and pulmonary inflammation in both human and animal models [60, 61]. As such, CHE models have been used to investigate the pulmonary effects of DE and WS, attempting to elucidate the pathways involved in these exposures and respiratory disease.

#### Neither DE nor WS are potent initiators of type 2 inflammation

Type 2 inflammation, which is associated with atopic diseases, is typically mediated via pathways involving mast cells, eosinophils, Th2 CD4 + cells, B cells, IgE, and cytokines such as interleukin (IL)-4, IL-5, and IL-13. These type 2 mechanisms are implicated in several pulmonary diseases, one of the most notable being asthma. As such, several CHE-DE and CHE-WS studies have examined the effect of these exposures on type 2 inflammation on the pulmonary system. Fractional exhaled nitric oxide (FeNO) is a noninvasive biomarker of airway eosinophilia [62, 63] and airway inflammation [64], and is often used in the clinical assessment and monitoring of asthma [65]. However, few studies have investigated the effect of WS and DE on FeNO in healthy, atopic, or asthmatic participants. In two separate CHE-WS studies conducted with 13 healthy participants each, FeNO was increased after exposure to WS at concentrations ranging from 150 to 275 µg/m^3^ PM_2.5_ for 180–240 min [66, 67], with this effect seen as far out as 43.5 h after the end of WS exposure [67]. However, two other CHE-WS studies, using similar sample sizes (19 and 14 participants respectively) found no significant difference in FeNO with WS exposure of similar duration and concentration [68, 69]. A small number of DE studies have also investigated the effect of DE exposure on FeNO. In two DE studies performed in healthy participants at 300 µg/m^3^ PM_2.5,_ there was no significant effect on FeNO after DE exposure, although these studies used shorter exposure durations of 30 min and 120 min, compared to the WS studies [70, 71]. A longer exposure duration may be needed to determine the effect of DE, as a different CHE-DE study utilizing a duration of 240 min demonstrated a concentration-dependent increase in FeNO when using lower concentrations of 20–150 µg/m^3^ PM_2.5_ [72]. This longer duration is more in line with those used in CHE-WS studies, which may explain why this positive effect on FeNO was primarily observed in these studies.

One CHE-WS study investigated the effect of WS on atopic participants. Riddervold et al. [73] found that there was no effect on FeNO after a 180-minute exposure to WS at concentrations of 200 and 400 µg/m^3^ PM_2.5_ in atopic participants without baseline airway hyperresponsiveness (AHR) [73]. Two CHE-DE studies have examined FeNO in asthmatic participants and found no significant difference between DE exposure for a comparatively shorter duration or at lower concentrations, 60 min at 300 µg/m^3^ PM_2.5_ [74] or 120 min at 100 µg/m^3^ PM_10_ [75]. Nonetheless, there is little evidence regarding the effect of both DE and WS exposure on FeNO, limiting the conclusions that can be drawn from this marker alone.

In addition to using FeNO as a surrogate, airway eosinophilia was also measured in some CHE-DE and CHE-WS studies. Three CHE-WS studies investigated eosinophils in bronchoalveolar lavage (BAL) and bronchial wash (BW) in healthy participants. Two of these studies found no effect of 180-minute WS exposures at concentrations of 200 µg/m^3^ PM_2.5_ in 19 participants [68] or 300 µg/m^3^ PM_1_ in 14 participants [69]. The third CHE-WS study, conducted in 14 subjects, demonstrated a significant increase in eosinophils in BAL (*p* = 0.02), but not BW, after a 120-minute exposure to WS at 400 µg/m^3^ PM_1_ [76]. CHE-DE studies using both lower (100 µg/m^3^ PM_10_) and similar (300 µg/m^3^ PM_10_) concentrations, as well as comparable sample sizes have also shown no significant effect of DE on eosinophils in BAL, BW, or bronchial submucosa in healthy participants, although these studies used shorter 60- or 120-minute exposure durations [75, 77, 78]. Notably, one CHE-DE study showed increased BAL but not BW eosinophils in healthy participants after 60 min of 300 µg/m^3^ PM_10_ DE exposure [79]. Taken together, the limited number of CHE-WS and CHE-DE studies has not confirmed any convincing effects of either WS or DE on airway eosinophils in healthy participants.

While no CHE-WS studies have directly measured airway eosinophils in atopic and/or asthmatic participants, some CHE-DE studies have been conducted in this population. In one study conducted in atopic participants with or without baseline AHR, exposure to 120 min of DE at 300 µg/m^3^ PM_2.5_ decreased BW eosinophils (*p* = 0.01), with no significant effect on eosinophils in BAL 48 h post-exposure [80]. Similarly, another CHE-DE study performed with asthmatic participants also revealed a small decrease in BW eosinophils (0.5% decrease, *p* < 0.05), with no differences observed in eosinophils in BAL or bronchial mucosa 6 h after 120 min of DE exposure at a lower concentration of 100 µg/m^3^ PM_10_ [81]. However, a third CHE-DE study performed in allergic rhinitics with the same exposure duration and concentration as Stenfors et al. [81] revealed that DE exposure did not affect eosinophils in BAL, the bronchial epithelium, or the bronchial submucosa at 18 h post-exposure [82]. Given the small number of studies, additional research is needed to elucidate the effect of DE and WS on airway eosinophils in both healthy individuals and atopic cohorts. Future studies could also consider exploring possible associations between airway eosinophils and systemic eosinophils; to date, only three CHE-DE studies [83–85] and no CHE-WS studies have investigated the effect of these exposures on systemic eosinophils.

Airway mast cells, another player implicated in type 2 inflammation, have been studied within the context of CHE-WS and CHE-DE models. In one CHE-WS study performed with 14 healthy, nonatopic participants, there was no effect on mast cells in BAL or BW after exposure to WS for 120 min at a concentration of 400 µg/m^3^ PM_1_ [76]. A different CHE-WS study completed with 19 healthy participants also revealed no effect on mast cells in BAL or BW after 180 min of WS exposure at 200 µg/m^3^ PM_2.5_ [68]. Similarly, while Muala and colleagues [69] found no difference in mast cells in BAL or BW after exposing 14 subjects to 180 min of WS at 300 µg/m^3^ PM_1_, they did find an increase (*p* < 0.05) in bronchial submucosal mast cells post-exposure. CHE-DE studies have also shown DE-induced increases in submucosal mast cells at lower PM concentrations and shorter exposure durations than relevant CHE-WS studies. In two separate CHE-DE studies conducted in 23 and 15 healthy participants respectively, a 120-minute exposure to DE at 100 µg/m^3^ PM_10_ significantly increased submucosal mast cells [75, 78]. A different CHE-DE study using an even shorter exposure duration of 60 min but a similar concentration of 300 µg/m^3^ PM_10_ to the aforementioned CHE-WS studies demonstrated a significant increase in bronchial submucosal mast cells, but not bronchial epithelial mast cells, in 15 healthy participants [77]. A CHE-DE study conducted with the same exposure duration and PM_10_ concentration as Salvi et al. [77] did not find an effect of DE exposure on bronchial submucosal or epithelial mast cells [79]. The two CHE-DE studies conducted by Behndig and colleagues [75, 78] discussed above also investigated mast cells in BAL and BW, and no effect of DE exposure was found. An older CHE-DE study conducted with a comparatively short exposure duration of 60 min also demonstrated no difference post-DE exposure in mast cells in BAL from 10 healthy participants, although the PM concentration was not reported in that study [86]. While no CHE studies have investigated the effect of WS on airway mast cells in atopic participants, two CHE-DE studies have investigated this topic. Larsson and colleagues [82] found no effect of 120 min of DE exposure at 100 µg/m^3^ PM_10_ on mast cells in BAL, BW, or bronchial submucosa in 14 participants with allergic rhinitis, although they did observe a significant decrease (*p* = 0.013) in bronchial epithelial mast cells. In the 2011 study conducted by Behndig and colleagues [75], DE exposure (120 min at 100 µg/m^3^ PM_10_) had no effect on mast cells in BW or the bronchial submucosa in 32 asthmatic participants. Overall, these limited number of studies imply that DE exposure induces an influx of mast cells into the bronchial submucosa in healthy participants, while there are too few CHE-WS studies to draw conclusions about the effect of WS on airway mast cells.

Taken together, the small number of CHE-WS studies conducted to date suggest WS is not a potent inducer of type 2 inflammatory pathways in the airways of healthy, nonatopic individuals, as indicated by FeNO, airway eosinophils, and airway mast cells. A comparatively greater number of CHE-DE studies have been performed in this area; notably, several CHE-DE studies, but no CHE-WS studies to date, have involved atopic/asthmatic participants. Based on these CHE-DE studies, there does not appear to be a clear effect of DE exposure on airway type 2 inflammation. Several CHE-DE studies have also explored other markers of eosinophilic/type 2 inflammation, such as IL-5, eotaxin-3, and eosinophil cationic protein (ECP), in BAL and BW samples, although no significant effects of DE on these markers have been reported [80, 81, 86]. Eotaxin-3, an eosinophil chemoattractant [87–89], has been found to be elevated in conditions characterized by type 2 inflammation such as asthma [90], atopic dermatitis [91], and eosinophilic esophagitis [92, 93]. Similarly, ECP, an eosinophil marker [94, 95] has also been shown to be increased in various atopic diseases [96–100]. To this end, future CHE-WS studies should consider including more diverse markers of type 2 inflammation, as well as including atopic and/or asthmatic participants, especially given the known association between atopic disease and type 2 inflammation.

#### DE interacts with allergen-induced type 2 inflammation in atopic participants

Several CHE studies have examined the effects of DE and allergen co-exposure on markers of eosinophilic and/or type 2 inflammation in atopic participants with and without baseline AHR. Ryu and colleagues [101] exposed participants to 120 min of DE at 300 µg/m^3^ PM_2.5_, followed by an inhalational allergen challenge. At 48 h post-exposure, exposure to FA and allergen increased eosinophils (*p* < 0.005), eotaxin-3 (*p* < 0.0001), and IL-5 (*p* < 0.0001) in BAL samples compared to FA and saline [101], demonstrating that allergen enhances levels of these mediators. However, co-exposure to the combination of DE and allergen had no effect on eosinophils, IL-5, or eotaxin-3 in BAL [101]. This abrogation of the allergen-provoked effect suggests that DE attenuates allergen-induced eosinophilic/type 2 inflammation. In another CHE study, instillation of an endobronchial allergen increased eosinophil counts in BW and BAL [80]. However, exposure to DE (120 min at 300 µg/m^3^ PM_2.5_) had no effect on eosinophils in BAL and decreased eosinophils in BW at 48 h post-DE exposure [80]. Similarly, DE alone did not affect levels of IL-5 or ECP in BAL, but allergen alone induced elevations in both markers [80]. Co-exposure to DE and allergen led to additive increases in eosinophils, IL-5, and ECP in BAL [80], suggesting DE augments allergen-induced type 2/allergic inflammation, in contrast to the implications of the findings of Ryu et al. [101]. Hosseini and colleagues [102] reported that while neither DE (120 min at 300 µg/m^3^ PM_2.5_) nor allergen alone affected endobronchial IL-4 staining, co-exposure to DE and allergen increased IL-4 at 48 h post-DE exposure, insinuating that DE may augment allergic inflammation, similar to Carlsten et al. [80]. The results from this small subset of CHE-DE studies imply that there is likely an interaction between exposure to allergens and DE with respect to eosinophilic and/or type 2 inflammation, though the details of this interaction should be further elucidated. However, to date, no CHE studies have investigated WS and allergen co-exposure, underscoring an area for future research.

#### DE induces airway neutrophilic inflammation in healthy populations, effect of WS is unclear

Neutrophilic inflammation has been implicated in the pathogenesis of both acute and chronic respiratory diseases, such as acute respiratory distress syndrome (ARDS), chronic obstructive pulmonary disease (COPD), bronchiectasis, and some asthma phenotypes [103–105]. Consequently, the influence of exposure to air pollution on pulmonary neutrophilic inflammation is of particular interest, as demonstrated by a number of CHE-DE and CHE-WS studies. In an early CHE-DE study, a 60-minute exposure to DE at 300 µg/m^3^ PM_10_ increased neutrophils in BW (increase of 1.29 cells × 10^4^ cells/mL, *p* = 0.009), bronchial submucosa (increase of 38.1 cells/mm^2^, *p* = 0.003), and bronchial epithelium (increase of 2.86 cells/mm, p 0.01) in 15 healthy subjects [77]. Additional studies have shown similar effects of DE on airway neutrophils. In two separate studies by Behndig and colleagues [75, 78], exposure to 120 min of DE at 100 µg/m^3^ PM_10_ significantly increased neutrophils in the bronchial submucosa and BW of healthy participants. Another study conducted with 25 healthy participants using similar exposure conditions revealed increased neutrophils in BW after DE exposure [81]. However, multiple studies performed at similar durations and analogous, if not higher, PM concentrations demonstrated no effect of DE on neutrophils in BAL or BW [85, 106]. One study conducted by Sehlstedt and colleagues [79] in 15 healthy participants at a shorter exposure duration of 60 min and concentration of 300 µg/m^3^ PM_10_ also showed no significant effect of DE on neutrophils in BAL, BW, the bronchial submucosa, or the bronchial epithelium.

Few CHE-WS studies have examined airway neutrophils thus far, highlighting an area for future investigation. In one CHE-WS study, conducted under comparable exposure conditions to those of Ryu et al. [85] (120 min of DE exposure at 300 µg/m^3^ PM_2.5_), 19 healthy participants were exposed to 180 min of WS at a concentration of 200 µg/m^3^ PM_2.5_ [68]. Like in Ryu et al. [85], there was no effect of WS exposure on neutrophils in BAL or BW at 24 h post-exposure [68]. Another CHE-WS study conducted in 14 healthy subjects with a similar duration (180 min) and concentration (300 µg/m^3^ PM_1_) also revealed no effect on levels of neutrophils in BAL, the bronchial submucosa, or the bronchial epithelium at 24 h after WS exposure [69]. However, exposure to a higher WS concentration of 500 µg/m^3^ PM_2.5_ increased neutrophils in BW and BAL at 20 h post-exposure in 10 healthy participants [107]. In contrast, a CHE-WS study conducted using comparable exposure conditions (120 min at 400 µg/m^3^ PM_1_) and sample size (14 subjects) revealed no effect of WS on BAL or BW neutrophils at 6 h post-exposure [76].

Multiple CHE-DE studies have also investigated the effects of DE exposure on sputum neutrophils. In a study conducted in healthy participants, the investigators found an increase in the percentage of sputum neutrophils (41% after DE compared to 32% after FA, *p* < 0.01) 4 h after exposure to 120 min of DE at 200 µg/m^3^ PM_10_ [108]. A different CHE-DE study using 60 min of DE exposure at 300 µg/m^3^ PM_10_ also revealed increased sputum neutrophil levels at 6 h post-DE exposure [109]. However, a study conducted by Carlsten and colleagues [110] revealed no effect of 120 min of DE exposure at 300 µg/m^3^ PM_2.5_ on sputum neutrophils at 28 h after exposure in a pooled analysis of participants with and without baseline AHR, and a decrease in sputum neutrophils after DE exposure in normally responsive participants. In the only CHE-WS study to date examining sputum neutrophils, healthy participants were exposed to 120 min of WS at a higher concentration of 500 µg/m^3^ PM_2.5_ compared to the above CHE-DE studies [111]. WS exposure increased sputum neutrophils (mean increase 10%, *p* = 0.0001) at 24 h post-exposure compared to pre-exposure [111]. Overall, evidence from CHE-DE studies suggests that DE exposure in the healthy population induces neutrophilia in the lower airways and likely in the upper airways. Given the limited number of CHE-WS studies, conclusions cannot be drawn on the relationship between WS and airway neutrophils.

Several CHE studies have used indirect markers of neutrophilic inflammation. Myeloperoxidase (MPO) is an enzyme primarily produced by polymorphonuclear neutrophils and is typically released in response to infection, although dysregulated release is implicated in various disease processes, including asthma, cystic fibrosis, and other pulmonary pathologies [103]. Another commonly used marker of neutrophilic inflammation is IL-8, a chemokine with potent neutrophil chemotactic and neutrophil-activating properties [112]. IL-8 has been implicated in the pathogenesis of several pulmonary diseases, such as ARDS, acute infection, and COPD [112–115]. Thus far, only two CHE-WS studies have examined airway MPO and one airway IL-8, although several CHE-DE studies have examined both of these markers. In a CHE-WS study conducted in healthy participants, Sehlstedt and colleagues [68] found no difference in MPO in BAL or BW after a 180-minute exposure to WS at 200 µg/m^3^ PM_2.5_. A different CHE-WS study exposed healthy participants to a higher concentration of WS at 300 µg/m^3^ PM_1_ for the same duration; these investigators found no difference in MPO levels in BAL with WS exposure and a slight decrease in MPO levels in BW (26.50 ng/mL (FA) vs. 17.80 ng/mL (WS), *p* = 0.019) [69]. Multiple CHE-DE studies conducted in healthy participants using shorter durations (60–120 min) and lower concentrations (100 µg/m^3^ PM) have mostly revealed no effect of DE exposure on airway MPO [75, 78, 81, 86, 106]. However, two of these CHE-DE studies have demonstrated evidence of a positive correlation between DE and MPO [75, 78].

In the only CHE-WS study to date in which airway IL-8 levels were measured, exposure to 120 min of WS at 500 µg/m^3^ PM_2.5_ did not affect BAL fluid concentrations of IL-8 in healthy participants [107]. However, in two CHE-DE studies conducted using the same exposure duration and lower concentration of PM (100 µg/m^3^ PM_10_), IL-8 in BAL and/or BW was increased post-DE exposure [78, 81]. Another CHE-DE study, conducted in 15 participants using a shorter duration of 60 min and a PM concentration of 300 µg/m^3^, similarly demonstrated increased levels of IL-8 mRNA in BW and bronchial tissue [116], further suggesting that DE is a greater inducer of neutrophilic inflammation than WS. Indeed, in another CHE-DE study conducted using the same exposure duration (120 min) but a lower PM concentration (300 µg/m^3^ PM_2.5_) than Ghio et al. [107], DE exposure increased neutrophil extracellular traps, another marker of neutrophil activation [117]. However, some CHE-DE studies have demonstrated no significant effect of DE exposure on airway IL-8 levels; these studies were conducted at similar durations (60–120 min), exposure concentrations (100–300 µg/m^3^ PM_10_), and sample sizes (23 or 15 healthy subjects) as those used in the studies above [75, 77]. Taken together, the currently available evidence from CHE studies indicates that DE exposure elicits neutrophilic inflammation in healthy populations, possibly to a greater degree than WS, with the considerable caveat that few CHE-WS studies have been conducted in this area.

#### DE does not induce neutrophilic inflammation in atopic or asthmatic populations, no studies of WS

A small number of CHE-DE experiments have examined airway neutrophils and associated markers in atopic and/or asthmatic participants. Two CHE-DE studies exposed asthmatic participants to DE for 120 min at a concentration of 100 µg/m^3^ PM_10_ [75, 81]. Stenfors and colleagues [81] found no significant effect of DE on neutrophils in BW, BAL, or the bronchial mucosa in 15 asthmatic subjects. Similarly, Behndig et al. [75]. did not find a difference in levels of neutrophils in bronchial submucosa or BW in 32 asthmatic participants after DE exposure [75]. A study conducted in 18 atopic participants with or without AHR also revealed no effect of DE on neutrophils in BAL or BW after 120 min of DE exposure at 300 µg/m^3^ PM_2.5_ [80]. One CHE-DE study examined airway neutrophils in 14 participants with allergic rhinitis, and the results revealed no effect of DE exposure (120 min, 100 µg/m^3^ PM_10_) on neutrophils in bronchial tissue, BW, or BAL [82]. However, one CHE-DE study showed a trend toward increased neutrophil counts after 120 min of DE exposure at 300 µg/m^3^ PM_2.5_ in participants with baseline AHR [110]. Several CHE-DE studies have also examined IL-8 and MPO as indirect markers of neutrophil activation in this population, with none of these studies demonstrating an effect of DE exposure on these surrogates [75, 80–82, 118].

Overall, the majority of these CHE-DE studies revealed no significant effect of DE exposure on airway neutrophilia in atopic/asthmatic populations. In contrast, CHE-DE studies conducted in healthy participants have demonstrated evidence that DE induces neutrophilic inflammation in this population. Perhaps the baseline airway inflammatory milieu in atopic populations is sufficiently variable that significance is not achieved. Alternatively, features of atopic populations could alter responses to DE challenge or may even have a protective effect, though specific mechanisms through which this may occur have yet to be elucidated. To date, no CHE-WS studies have explored the relationship between WS exposure and airway neutrophils in these populations, revealing another area for future investigation.

#### Possible synergistic effect of DE and allergen co-exposure on neutrophilic airway inflammation

A subset of CHE-DE studies examined neutrophilic inflammation in participants with atopy, both with and without baseline AHR. Carlsten and colleagues [80] demonstrated an additive effect of DE (120 min at 300 µg/m^3^ PM_2.5_) on endobronchial allergen-provoked increases in neutrophils in BAL, as well as levels of IL-8 in BAL and BW at 48 h post-DE exposure, although exposure to DE alone was not correlated with any changes. Hosseini and colleagues [102] reported no effect of DE (120 min at 300 µg/m^3^ PM_2.5_) or allergen alone on bronchial submucosal neutrophil elastase, a neutrophil marker, at 48 h post-DE exposure. However, the combination of DE and allergen significantly increased (*p* = 0.03) neutrophil elastase [102], suggesting that there may be a synergistic effect between DE and allergen co-exposure. Another CHE-DE study also demonstrated that exposure to DE (120 min of DE at 300 µg/m^3^ PM_2.5_) may enhance endobronchial allergen-induced increases in MPO in both BAL and BW [118]. However, no similar co-exposure CHE experiments have been conducted with WS, which may be of interest for future studies.

#### DE does not clearly induce lymphocytic inflammation in the airways, unclear effect of WS

Dysregulation of lymphocytic inflammation has been implicated in a host of respiratory diseases [119–122], accentuating interest in the effect of air pollution on these mediators of airway inflammation. Only two CHE studies thus far have examined the effect of WS exposure on airway lymphocytes in healthy populations. Muala and colleagues [69] exposed 14 healthy participants to 180 min of WS at 300 µg/m^3^ PM_1,_ revealing decreased total lymphocytes in BAL and BW, and a decrease in CD4 + and CD8 + cells in BAL fluid at 24 h after WS exposure. However, they also found increased CD3 + lymphocytes and CD8 + cells in the bronchial submucosa and/or epithelium [69]. In a different CHE-WS study conducted in 19 healthy subjects, there was no effect of exposure to 180 min of WS at 200 µg/m^3^ PM_2.5_ on lymphocytes in BAL or BW [68]. Similarly, other CHE-DE studies conducted with similar sample sizes and a slightly shorter exposure duration (120 min) at various concentrations (100–300 µg/m^3^ PM) also demonstrated no significant effect of DE exposure on lymphocytes in BAL, BW, or the submucosa from 6 to 24 h post-exposure [78, 85]. No effect of DE on airway lymphocytes was found as well in other CHE-DE studies conducted at a shorter duration of 60 min [79, 86]. However, two CHE-DE studies have demonstrated some evidence of increased airway lymphocytes at 6 h post DE exposure [77, 81].

While no CHE-WS studies have examined airway lymphocytes in asthmatic or atopic participants, these populations have been included in some CHE-DE studies. The majority of these studies revealed no significant effect of DE exposure on lymphocytes in BAL or BW [80–82, 85]. While some of these studies did examine specific lymphocyte subsets, a considerable portion of these studies examined total lymphocytes only. Notably, two CHE-DE studies involving atopic participants with and without baseline AHR investigated the effects of DE and allergen co-exposure on lymphocyte populations. In the first study, total lymphocyte, CD8 + T cell, and CD4 + T-cell counts were not affected by DE exposure (120 min at 300 µg/m^3^ PM_2.5_), whereas total lymphocyte counts were decreased by DE at 48 h post-exposure (1.1 vs. 0.2 cells ×10^3^/mL, *p* = 0.01) [80]. Co-exposure to DE and allergen did not have any additional effects on total lymphocytes but was shown to have synergistic effects on CD4 expression in helper T cells [80]. In another study, endobronchial instillation of allergen increased the staining intensity of the plasma cell marker CD138 [102]. Allergen in combination with DE (120 min at 300 µg/m^3^ PM_2.5_) increased CD138 staining more than DE alone or allergen alone [102]. Co-exposure to allergen and DE also increased CD4 staining compared to FA and saline control (*p* = 0.04) [102], further suggesting interplay between these two exposures. Previous literature has shown that individual lymphocyte subsets are differentially involved in the pathogenesis of specific pulmonary diseases [119, 121, 122], highlighting the need for CHE-DE and CHE-WS studies to separately examine major lymphocyte populations. In general however, few CHE-WS studies have been conducted in this area, underscoring yet another focus for future investigation.

#### Neither WS nor DE affect airway macrophages

Evidence from CHE studies seem to reveal no effect of WS or DE exposure on airway macrophages in healthy populations. In three CHE-WS studies using 14 or 19 healthy subjects, no changes to levels of macrophages in BAL or BW were observed with WS exposure for 120–180 min at concentrations of 200–400 µg/m^3^ PM [68, 69, 76]. CHE-DE studies conducted using sample sizes of 15 subjects and a shorter exposure duration of 60 min, but a similar concentration of 300 µg/m^3^ PM_10_ similarly demonstrated no effect of DE on macrophages in BAL, BW, or bronchial tissue [77, 79]. In another CHE-DE study using an exposure duration of 120 min, comparable to the CHE-WS study but a lower concentration of 100 µg/m^3^ PM_10_, DE had no significant effect on macrophages in BAL or BW in 15 healthy participants [78]. One older CHE-DE study demonstrated increased macrophages in BAL after a short 60-minute exposure, with the caveat of an unknown exposure concentration [86]. Two other CHE-DE studies conducted in atopic populations revealed no effect of DE exposure on macrophages in BAL and BW [80, 82]. No CHE-WS studies have been conducted in atopic populations thus far, indicating a potential area for further exploration.

#### CHE studies have explored potential effects of DE and WS on other proinflammatory markers in the airways

CHE-DE and CHE-WS studies have examined a variety of other proinflammatory markers in the airways, including IL-6, matrix metalloproteinase-9 (MMP-9), tumor necrosis factor-alpha (TNF-α), intracellular adhesion molecule-1 (ICAM-1), and IL-1b. Ghio and colleagues [107] conducted a CHE-WS study in which 10 healthy participants were exposed to 120 min of WS at 500 µg/m^3^ PM_2.5_. They found no significant effect of WS exposure on concentrations of IL-6 in BAL or BW at 20 h post-exposure [107]. Similarly, a CHE-WS study performed in 14 healthy participants by Muala and colleagues [69] revealed no effect of WS exposure (180 min at 300 µg/m^3^ PM_1_) on levels of IL-6 in BAL or BW at 24 h post-exposure. In contrast, CHE-DE studies also conducted using exposure durations of 120 min, but lower PM concentrations (100 µg/m^3^ PM_10_) have shown increased IL-6 levels in BW at 6 h [81] and 18 h [75] post-exposure in 25 and 23 healthy participants respectively. However, a different CHE-DE study using similar exposure duration and PM concentration did not demonstrate an effect of DE on IL-6 levels in BAL or BW at 18 h post-exposure in 15 healthy participants [78]. The above study by Ghio and colleagues [107] also investigated other proinflammatory markers, including IL-1b and TNF-α, in BAL fluid samples, revealing that WS exposure had no effect on either parameter at 20 h post-exposure. Similarly, one CHE-DE study revealed no effect of DE (60 min at 300 µg/m^3^ PM_10_) on IL-1b or TNF-α mRNA expression in BW or bronchial tissue [116]. Other CHE-DE studies conducted with similar exposure durations to those of Ghio et al. [107] (120 min) but lower PM concentrations (100–300 µg/m^3^ PM) have also demonstrated no significant effect of DE on TNF-α levels in BAL or bronchial tissue [80, 81].

ICAM-1, a mediator of several inflammatory pathways [123], is another marker that has been examined in CHE studies. In a CHE-WS study conducted by Muala and colleagues [69], 14 healthy participants were exposed to 180 min of WS at 300 µg/m^3^ PM_1_. At 24 h post-exposure, WS decreased ICAM-1 in BW (35.20 ng/mL (air) vs. 18.30 ng/mL (WS), *p* = 0.028), but did not significantly affect ICAM-1 in BAL [69]. Multiple CHE-DE studies using similar sample sizes, slightly shorter exposure durations of 120 min, and PM concentrations ranging from 100 to 300 µg/m^3^ PM have also shown no effect of DE on ICAM-1 in bronchial tissue, BAL, or BW in healthy participants [75, 78, 81, 85, 106]. Two CHE-DE studies using less comparable durations of 60 min but similar PM concentrations (300 µg/m^3^ PM_10_) and numbers of participants (15) also demonstrated no effect of DE on airway ICAM-1 [77, 79]; however, one of these studies did show a positive correlation between DE exposure and bronchial tissue ICAM-1 expression [77].

Muala and colleagues [69] also examined another proinflammatory marker, MMP-9, in their CHE-WS study. They found no effect of WS on MMP-9 levels in BAL but a small decrease in BW (13.40 ng/mL (air) vs. 6.60 ng/mL (WS), *p* = 0.006) [69]. Another CHE-WS study demonstrated no significant influence of WS exposure (180 min at 200 µg/m^3^ PM_2.5_) on MMP-9 levels in BAL and BW of 19 healthy volunteers [68]. In a CHE-DE study, MMP-9 was not affected by DE exposure (120 min at 300 µg/m^3^ PM_2.5_) [85]. While the majority of these proinflammatory markers were not affected by DE or WS exposure in these CHE studies, it should be noted that this may be an unfair comparison. The CHE-DE studies tended to use shorter exposure durations and/or lower PM concentrations than the CHE-WS studies. As such, future studies utilizing more similar exposure conditions could provide a more equitable comparison of the effects of WS and DE. It should also be noted that only a small number of CHE-WS studies have explored airway inflammation in general, greatly limiting any conclusions that can be drawn.

#### Few CHE studies have examined the effect of WS or DE on airway SP-D, SP-A, and CC16

Surfactant protein D (SP-D), surfactant protein A (SP-A), and club cell protein 16 (CC16) are endogenous proteins expressed in the lung that play important roles in pulmonary immunity and homeostasis. SP-D and SP-A participate in host lung defense via direct binding and subsequent enhanced clearance of pathogens but also through modulatory interactions with various immune cells [124–126]. Both SP-D and SP-A have been implicated in various chronic pulmonary diseases, such as COPD, asthma, and interstitial lung disease [126–130]. Similarly, dysregulation of CC16, a protein secreted by club cells, has been associated with various respiratory conditions [131–133]. CC16 has been shown to have a protective effect in COPD [132, 134–136], attracting interest in potential therapeutic applications [137, 138]. Only two CHE-DE studies to date have investigated the influence of DE exposure on these mediators. In the first study, 14 atopic participants with or without baseline AHR were exposed to DE or particle-depleted DE (PDDE) and inhaled allergen (120 min at 300 µg/m^3^ PM_2.5_) and subsequently underwent bronchoscopy at 48 h after completion of each DE exposure [101]. The authors demonstrated an allergen-induced increase in SP-D levels in BAL, an effect that was attenuated by DE but not by particle depletion of DE [101]. SP-D has been shown to aid in the clearance of allergens from the lung [139]; thus, these results from Ryu et al. [101] imply a possible detrimental effect of DE on the pulmonary response to allergens. There was no effect of DE exposure in the context of allergen exposure on CC16 or SP-A levels in BAL in this study [101]. In a different CHE-DE study, 18 atopic participants with or without AHR were exposed to DE (120 min at 300 µg/m^3^ PM_2.5_), then underwent endobronchial instillation of allergen and saline control into contralateral lungs [118]. At 48 h post-exposure, there was no effect of DE compared to FA on SP-D levels in BAL or BW, or on CC16 levels in BW [118]. However, DE decreased CC16 levels in BAL (*p* = 0.04), also implying a detrimental impact of DE on this protective protein [118]. While no CHE studies have explored the effects of WS on pulmonary SP-D or SP-A, one CHE-WS study investigated the effect of WS (180 min at 300 µg/m^3^ PM_1_) on CC16 levels in BAL and BW in 14 healthy participants, finding no significant effect of WS [69]. Given the importance of these airway proteins as players in lung defense mechanisms and as disease markers, future research should be conducted on the interplay of these proteins with DE and WS as models of common air pollution sources.

#### DE may exacerbate live attenuated influenza virus-induced allergic inflammation, unclear effect of WS

Two CHE-DE studies have examined the interaction between DE exposure, atopy, and response to viral challenge. In these studies, 16 or 11 participants with allergic rhinitis were exposed to either 120 min of DE at 100 µg/m^3^ PM or FA, followed by intranasal instillation of live attenuated influenza virus (LAIV) [140, 141]. Noah and colleagues [140] also included 16 participants without allergic rhinitis. Nasal lavage fluid samples were then collected between 1 and 9 days [140] or 1 and 10 days post-exposure [141]. DE exposure increased eotaxin-1 and ECP at up to 10 days post-exposure [140, 141], suggesting that DE in the context of LAIV exacerbates allergic inflammation over a fairly prolonged period. This effect of DE on eotaxin-1 and ECP was also shown to interact with allergy status, implying atopic individuals may be more susceptible to DE-induced allergic inflammation [140]. DE also decreased IP-10, a marker of NK cell activation, suggesting that DE in this context may reduce NK cell clearance of eosinophils [141]. One CHE-WS study, conducted in 39 healthy participants, also examined the effect of WS and LAIV co-exposure on inflammatory marker levels in nasal lavage fluid collected at 1 and 2 days post-exposure [142]. The authors found no effect of WS and LAIV co-exposure on ECP or other allergic inflammatory markers, including eotaxin-3, IL-5, and IL-13 [142]. Interestingly, this study demonstrated the suppressive effect of WS on IP-10 [142], suggesting that NK cells may be shared mediators of immune responses to WS and DE exposure. Future research could build on these studies, elucidating the interactions between allergic inflammation, viral infection, and exposure to DE or WS.

#### Acute exposure to DE or WS does not affect pulmonary function

Short-term exposure to DE in CHE studies has not been shown to affect lung function in healthy participants, as measured by pulmonary function testing. Multiple CHE-DE studies using exposures ranging from 60 to 120 min and 100–300 µg/m^3^ PM have demonstrated no significant change in pre- to post-exposure forced expiratory volume in 1 s (FEV_1_) or forced vital capacity (FVC) [70, 72, 77, 79, 81, 84, 106, 108, 110, 143–150]. However, most of those studies were conducted immediately post-exposure, although some have measured these parameters up to 28 h after exposure completion. Similarly, no effect of DE exposure on FEV_1_ or FVC has been shown in participants with baseline lung disease, such as asthma [75, 110, 150, 151] or COPD [144]. However, one CHE study demonstrated a small but significant decrease in FEV_1_ (3.3% decrease in predicted FEV_1_ from baseline, *p* = 0.04) with 60 min of DE exposure at 300 µg/m^3^ PM_2.5_ [74]. Interestingly, in a CHE study examining the effects of DE (120 min at 300 µg/m^3^ PM_2.5_) and endobronchial allergen co-exposure in atopic participants with and without baseline AHR, co-exposure to DE and allergen decreased FEV_1_ at 2 h post-exposure only in participants with gene variants of glutathione S-transferase T1 [152], suggesting that genetics may also play a role in the response to environmental co-exposure.

CHE-WS studies have also demonstrated no significant effect of WS exposures ranging from 45 to 180 min and 200–500 µg/m^3^ PM on FEV_1_ or FVC in healthy participants at up to 24 h post-exposure [68, 69, 73, 107, 153, 154]. CHE studies in healthy participants have also revealed no effect of DE on the FEV_1_/FVC ratio [72, 144, 148]. WS has also been shown to not affect the FEV_1_/FVC ratio in healthy participants [69, 107, 153, 154]. Thus far, no CHE-WS studies have examined lung function in subjects with asthma, highlighting a gap in the literature.

#### DE increases airway responsiveness in asthmatics, no evidence from CHE-WS studies

Several CHE-DE studies have examined the effects of DE on airway responsiveness. The provocative concentration of methacholine needed to cause a 20% decrease in FEV_1_ (PC_20_) is often used to quantify airway responsiveness, with a cutoff of PC_20_ *≤* 8 mg/mL typically used as the criteria for defining AHR [155]. Hussain and colleagues [74] exposed participants with previous diagnosis of asthma to 60 min of DE at 300 µg/m^3^ PM_2.5_, then performed methacholine challenge testing 24 h post-exposure. Notably, not all participants had baseline AHR as determined by methacholine challenge [74]. They found that DE exposure significantly reduced mean PC_20_ (*p* = 0.012) in all subjects, reduced PC_20_ in those with baseline AHR (*p* = 0.078), and increased the number of individuals who tested methacholine positive (i.e. PC_20_ *≤* 8 mg/mL) (3 subjects post FA versus 7 subjects post DE) [74]. Another CHE-DE study examined airway responsiveness in participants both with and without baseline AHR after exposure to 120 min of DE at 300 µg/m^3^ PM_2.5_ [110]. The authors found no effect of DE on airway responsiveness (as determined by methacholine testing) in subjects without baseline AHR, but demonstrated that DE significantly increased airway responsiveness in those with baseline AHR [110]. In a third study involving 14 participants all with baseline AHR, exposure to 60 min of DE at 300 µg/m^3^ PM_10_ significantly decreased methacholine PC_20_ at 24 h post-exposure [151]. However, one CHE-DE found no effect of DE on airway responsiveness in 32 asthmatic subjects in both a separate analysis of those with baseline AHR and a pooled analysis of all asthmatics with or without baseline AHR [75]. However, methacholine testing was conducted at a much later timepoint (40 h post-exposure) and with a lower DE concentration of 100 µg/m^3^ PM_10_ in comparison to the 300 µg/m^3^ PM used in previous studies [75]. Likewise, no interaction between DE and methacholine response in healthy, non-asthmatic individuals was shown in two different CHE-DE studies using concentrations of 200 µg/m^3^ PM_10_ or 20–150 µg/m^3^ PM_2.5_ [72, 108]. No CHE-WS studies have investigated the effect of WS on airway responsiveness to date, although epidemiological studies have demonstrated the detrimental effects of WS on asthma symptoms and severity [156–159]. As such, experimental evidence from CHE models could serve to better elucidate the interaction between airway responsiveness and WS exposure, highlighting an important focus for future research.

Several older studies have proposed a correlation between airway eosinophilia and airway responsiveness in asthmatics [160–164]. As previously discussed, two CHE-DE studies have shown an apparent DE-related decrease in airway eosinophils in participants with atopy and/or baseline AHR [80, 81]; however, this finding contradicts the observed DE-induced exacerbation of airway responsiveness in those with baseline AHR [74, 110, 151]. Evidence from newer studies suggests that airway eosinophils may not be the main causative agent of increased airway reactivity [165–168], implicating other mechanisms. Based on the limited number of relevant CHE-DE studies, it is also difficult to clearly delineate the relationship between DE and airway eosinophilia. As such, further investigations are needed to better characterize the interplay between airway eosinophilia, airway responsiveness, and air pollutant exposure.

### Systemic inflammation

#### Molecular markers of systemic inflammation

Observational studies have demonstrated associations between exposure to traffic-related or biomass burning-related air pollution and systemic inflammation [169–173]. As such, multiple CHE studies have explored the relationships between exposure to DE or WS and systemic inflammatory markers. Several CHE-WS studies conducted in healthy participants using mostly 180-minute exposures (ranging from 120 to 240 min) and concentrations of 200–300 µg/m^3^ PM (ranging from 150 to 500 µg/m^3^ PM) have demonstrated no effect of WS on circulating proinflammatory markers such as IL-6 [69, 107, 174], IL-8 [69, 107], VCAM-1 [174], ICAM-1 [69, 174], TNF-α [69, 107, 174, 175], or CRP [174, 175] at timepoints ranging from immediately to 44 h after exposure completion. CHE-DE studies have investigated similar markers in healthy participants as well, similarly revealing no effects of DE exposure on serum IL-6 [108, 148, 176–179], IL-8 [84, 180], VCAM-1 [83], ICAM-1 [68, 148, 176, 179, 181], TNF-α [108, 148, 176–179], or CRP [176–178, 181–184]. No effect of DE (120 min at 200 µg/m^3^ PM_2.5_) on many of these markers was demonstrated in a CHE study conducted in participants with metabolic syndrome [185]. In comparison to their WS counterparts, these CHE-DE studies generally utilized shorter durations of 60–120 min (ranging from 30 to 180 min), but analogous PM concentrations of 200–300 µg/m^3^ (ranging from 20 to 350 µg/m^3^) at timepoints spanning from immediately to 24 h post-exposure. Additionally, in contrast to those of WS, some CHE studies have shown DE-induced increases in the levels of these specific proinflammatory markers in healthy cohorts. In a CHE-DE study conducted by Tousoulis and colleagues [186], healthy smokers and nonsmokers were exposed to 120 min of DE at a comparatively low concentration of 25 µg/m^3^ PM_2.5_, with blood samples obtained immediately after exposure and at 24 h post-exposure. DE significantly increased CRP and fibrinogen, another positive acute-phase reactant, even after accounting for smoking status in a linear mixed model analysis [186]. Interestingly, there was a greater increase in CRP in smokers than in nonsmokers [186]. A different CHE-DE study showed DE exposure (60 min at 300 µg/m^3^ PM_10_) increased serum TNF-α and IL-6 levels (*p* = 0.02 for both) at 24 h post-exposure [181]. Mookherjee and colleagues [184] conducted a CHE-DE study with a longer 240-minute exposure at a concentration of 150 µg/m^3^ PM_2.5_, which is comparatively lower than most CHE-WS studies, and found that exposure to DE increased serum IL-6 concentration at 24 h (*p* < 0.01). Similarly, another CHE-DE study demonstrated an increase in serum IL-6 mRNA expression after 120 min of exposure to DE at a PM concentration of 200 µg/m^3^ PM_2.5_ DE [187]. A different CHE-DE study using conditions (180 min at 300 µg/m^3^ PM_1_) comparable to those used in WS experiments demonstrated a trend toward increased serum IL-6 (*p* = 0.07) at 20 h post-exposure [84].

There is some evidence that WS may also induce systemic inflammation. In a CHE-WS study conducted by Barregard and colleagues [175], healthy participants were exposed to WS at a concentration of 240–280 µg/m^3^ PM_1_ for 240 min, with blood sampled at 0, 3, and 20 h post-exposure [175]. The serum amyloid A concentration, a positive acute phase reactant, was significantly increased by WS exposure at all three time points (*p* < 0.01 for all) [175]. However, WS had no significant effect on other serum proinflammatory markers, such as CRP, fibrinogen, or TNF-α [175]. Interestingly, WS decreased serum IL-6 compared to clean air at 3 hours post-exposure [175]. A different CHE-WS study, however, demonstrated no effect of WS (180 min at 150–200 µg/m^3^ PM_2.5_) at timepoints ranging from 4 h to 44 h post-exposure [174]. Overall, there is some evidence from CHE studies suggesting that DE may induce systemic inflammation, while there is more limited evidence for a proinflammatory effect of WS. Notably, the CHE-DE studies tended to use shorter durations and less concentrated PM levels than did the WS studies, further suggesting a greater inflammatory influence of DE.

#### No significant effect of neither DE nor WS on total circulating leukocytes

CHE studies have also investigated the effect of DE and WS exposure on circulating leukocytes. CHE-WS studies have demonstrated no effect of WS exposures ranging from 60 to 240 min at concentrations of 150–115 µg/m^3^ PM on circulating total leukocyte counts in healthy participants at timepoints beginning from immediately to up to 44 h post-exposure [107, 174, 175, 188]. Similarly, with one exception, CHE-DE studies conducted in healthy participants have not demonstrated a significant effect of DE exposures ranging from 20 to 120 min at concentrations of 200–350 µg/m^3^ PM on total leukocyte counts at timepoints ranging from immediately to up to 24 h after exposure completion [83, 85, 145, 176–178, 181, 185, 189–191]. Only one CHE-DE study revealed an increase in total leukocyte count at 20 h post-DE exposure, although this study used a longer exposure duration of 180 min and a concentration of 300 µg/m^3^ PM_1_ [84].

#### Some evidence for a pro-neutrophilic effect of DE, limited evidence for WS

What is likely more illuminating are the effects of DE and WS on specific leukocyte populations, namely neutrophils. Several CHE-DE studies have revealed no significant effect of DE exposure (20–180 min, 200–350 µg/m^3^ PM) on levels of circulating neutrophils at timepoints ranging from immediately to up to 24 h post-exposure in healthy participants [83–85, 176–178, 181, 185, 190, 192]. However, two CHE studies have demonstrated increased neutrophils post-DE exposure. Stiegel and colleagues [193] exposed 15 healthy participants to 120 min of DE at 300 µg/m^3^ PM and found DE significantly increased both the absolute number (*p* = 0.028) and percentage (*p* = 0.0004) of neutrophils immediately after the end of exposure, though with the caveat of there being a significant increase in the percentage of neutrophils (*p* = 0.001) after clean air exposure at this timepoint [193]. A different CHE-DE study also demonstrated an increase in neutrophil count (1.08-fold increase compared to pre-exposure, *p* < 0.05) 18 h after exposure to 120 min of DE at 200 µg/m^3^ PM in 6 subjects, in comparison to baseline values measured prior to DE exposure [182]. However, the same study demonstrated no effect of DE at a higher concentration of 300 µg/m^3^ PM [182]. Several CHE-DE studies have also investigated the effect of DE on circulating neutrophils in different populations. One CHE-DE study explored the effects of DE exposure (60 min at a concentration of 300 µg/m^3^ PM_10_) in men with stable coronary artery disease and a prior history of myocardial infarction and demonstrated no difference in neutrophil counts at 6 and 24 h post-DE exposure [194]. Two CHE-DE studies investigated the effects of DE (120 min at a concentration of 300 µg/m^3^ PM_2.5_) in never smokers and ex-smokers with or without COPD [85, 117]. While Ryu and colleagues [85] revealed no effect of DE on neutrophil counts at 24 h post-exposure in any of the groups, Wooding and colleagues [117] reported that DE decreased the percentage of neutrophils (− 6.2%, *p* = 0.04) at 24 h post-exposure. Interestingly, Wooding and colleagues [117] also demonstrated increased neutrophil expression of activation surface markers in participants with COPD in comparison to never-smokers, suggesting a possible greater pro-neutrophilic effect of DE in those with COPD.

Very few CHE studies have investigated the effect of WS on peripheral blood neutrophils thus far. In the first study conducted by Ghio and colleagues [107], healthy participants exposed to WS (120 min at 500 µg/m^3^ PM_2.5_) were found to have increased absolute neutrophil counts (17% increase, *p* < 0.01) and percentage neutrophils (11% increase, *p* = 0.04) at 20 h post-exposure. A different CHE-WS study, conducted by Peters and colleagues [195], investigated serum MPO concentration, an indirect marker of neutrophil activity, and found levels of MPO were increased immediately after 90-minute exposures to WS at 250 and 500 µg/m^3^ PM_2.5_, further implying a pro-neutrophilic effect of WS. However, the only other CHE-WS study demonstrated no effect of a 60-minute exposure at a considerably higher PM concentration of 1100 µg/m^3^ PM_1_ on circulating neutrophils in healthy male firefighters from 2 to 24 h post-exposure [188]. The limited number of studies conducted in this area greatly restricts the conclusions that can be drawn, highlighting another focus for future research.

#### Neither DE nor WS affect circulating lymphocytes

Only two CHE studies have examined the effects of WS on peripheral blood lymphocytes. Hunter and colleagues [188] exposed 16 healthy male firefighters to 60 min of WS at a concentration of 1100 µg/m^3^ PM_1_. They found no significant effect of WS on lymphocytes at 2, 6, or 24 h post-exposure [188]. Similarly, in a different CHE-WS study, there was no clear effect of WS exposure (120 min at 500 µg/m^3^ PM_2.5_) on lymphocytes in 10 healthy participants immediately or at 20 h post-exposure [107]. In another CHE-WS study, peripheral blood mononuclear cells were collected from 14 healthy participants after four 120-minute exposures to WS at 500 µg/m^3^ PM, and no effect of WS on the degree of induced T-cell proliferation was detected in vitro [196]. A different CHE-WS also demonstrated no effect of WS (180 min at 300 µg/m^3^ PM_1_) on CD3+, CD4+, or CD8 + lymphocytes [69]. However, the small number of CHE-WS studies makes it difficult to clearly delineate the relationship, or lack thereof, between WS and circulating lymphocytes.

CHE-DE studies have predominantly demonstrated no effect of DE exposures (ranging from 21 to 120 min at 200–350 µg/m^3^ PM) on circulating lymphocytes in healthy participants at timepoints spanning immediately to 24 h after exposure completion [83, 84, 177, 185, 190, 192]. These DE exposures are of largely shorter duration and have lower concentrations than the WS exposures discussed above. Interestingly, one CHE-DE study conducted in 6 healthy participants demonstrated a 30% decrease (*p* < 0.05) in circulating lymphocytes 30 min after 120-minute, 100 µg/m^3^ PM DE exposure compared to baseline measurements obtained prior to DE exposure [182]. The same study, however, revealed no effect of higher DE concentrations (200 and 300 µg/m^3^ PM) on lymphocytes at 30 min and 18 h post-exposure [182], suggesting that the effect observed with 100 µg/m^3^ PM may have been observed by chance. Another CHE-DE study showed DE decreased peripheral blood lymphocytes (*p* < 0.0001) immediately after a 120-minute, 300 µg/m^3^ PM exposure; however, the same study also demonstrated a significant reduction in lymphocytes (*p* < 0.0025) after exposure to clean air at the same timepoint [193].

Some CHE-DE studies have also included participants with various chronic diseases. Vieira and colleagues [192] examined heart failure patients in their CHE study and found no effect of DE (21 min at 300 µg/m^3^ PM_2.5_) on circulating lymphocytes in this cohort. A different CHE-DE study examined the relationship between DE exposure (120 min at 300 µg/m^3^ PM_2.5_) and circulating lymphocytes in ex-smokers with COPD, ex-smokers without COPD, and healthy never-smokers [85]. In a pooled analysis of all three groups, the investigators found a significant increase in lymphocytes at 24 h post-exposure [85]. While this study revealed no significant interaction effect of COPD [85], this is the only CHE-DE study to date that has demonstrated a significant increase in lymphocytes. However, Yamamoto and colleagues [197] conducted another notable CHE-DE study in which asthmatic individuals were exposed to 120 min of DE at 300 µg/m^3^ PM_2.5_. At 4 h after exposure, there was no significant effect of DE exposure on circulating lymphocytes, suggesting the presence of an interaction between DE and asthma unlike DE and COPD, or perhaps differential effects of DE on separate lymphocyte subsets. A direction for future study could be to explore the effects of DE and WS exposure on individual lymphocyte populations, given the known heterogeneity in their involvement across various diseases. Future CHE studies could also include more participants with chronic conditions to delineate the complex relationships between DE and WS exposure and disease pathophysiology.

#### Unclear effect of WS on monocytes, CHE-DE studies suggest weak or no effect of DE

Only one CHE-WS study has examined the influence of WS on circulating monocytes [107]. In this study, 10 healthy participants were exposed to WS for 120 min at a concentration of 500 µg/m^3^ PM_2.5_, with blood collected immediately and at 20 h post-exposure [107]. The investigators found no effect of WS on circulating monocytes [107]. Similarly, a number of CHE-DE studies have shown no significant effects of DE exposure (21–120 min at 200–350 µg/m^3^ PM) on peripheral blood monocytes [83, 85, 177, 185, 192, 193]. One CHE-DE study reported that DE exposure over a longer 180-minute period and a concentration of 300 µg/m^3^ PM_1_ increased peripheral monocytes [84]. Another CHE-DE study also demonstrated an increase in monocytes after 120 min of DE at 100 µg/m^3^ PM when compared to baseline values obtained pre-exposure, although this study revealed no effect of DE at higher concentrations of 200 and 300 µg/m^3^ PM [182]. Monocytes were not shown to be affected by DE in ex-smokers with or without COPD (DE exposure of 120 min at 300 µg/m^3^ PM_2.5_) [85] or in participants with heart failure (DE exposure of 21 min at 300 µg/m^3^ PM_2.5_) [192]. Overall, while there are not enough CHE-WS studies to reach definitive conclusions, evidence from CHE-DE studies suggests that DE does not strongly induce monocyte-mediated inflammation. However, it is possible that previous CHE-DE studies have not used long enough durations or high enough PM concentrations, given that one CHE-DE study using a longer exposure duration did demonstrate an increase in monocytes [84].

#### Few studies have examined the influence of WS and DE on serum SP-A, SP-D, and CC16

SP-A and SP-D are surfactant proteins produced in the lungs; however, the serum levels of these proteins have also been shown to correlate with the severity and/or progression of various pulmonary diseases, such as COVID-19 [198–200], interstitial lung disease [201–204], and COPD [205, 206]. Interestingly, several studies have proposed that SP-A and SP-D are biomarkers of PM exposure [207]. Two CHE-WS studies have examined the influence of WS on serum SP-D and SP-A in healthy participants using exposure durations of 90–180 min and concentrations ranging from 150 to 500 µg/m^3^ PM_2.5_ [67, 208]. Both of these studies demonstrated no effect of WS on serum SP-D or SP-A at timepoints spanning immediately to 44 h post-exposure to PM_2.5_ [67, 208]. One CHE-DE study also conducted in healthy participants using 180 min of DE exposure at 300 µg/m^3^ PM_1_ similarly reported no effect of DE on SP-D immediately or at 6 h post-exposure [84].

CHE studies have also investigated the effect of WS or DE exposure on these markers in atopic participants. In a CHE-WS study, 20 atopic participants were exposed to WS for 180 min at 200 and 400 µg/m^3^ PM, with blood sampled immediately, 6 h, and 20 h after exposure completion [209]. There was no effect of WS on serum SP-D or SP-A at any timepoint tested [209]. In two CHE-DE studies, atopic participants with or without AHR were exposed to DE (120 min at 300 µg/m^3^ PM_2.5_), inhaled allergen [101], or endobronchial allergen instillation in one lung [118]. In peripheral blood collected at 4, 24, and 48 h post-exposure, there was no clear effect of DE on SP-D [101, 118] or on SP-A [101], suggesting that co-exposure to DE and allergen had no effect on these markers. Taken together, the evidence from CHE studies has not demonstrated clear effects of DE or WS on serum SP-D or SP-A in either healthy or atopic populations. The implications of these findings are unclear, given the still emerging role of these proteins as markers in various respiratory diseases. However, as research elucidates the prognostic value of serum SP-D and SP-A concentrations, further exploration of these markers with respect to DE and WS exposure may be of interest for future CHE studies.

Circulating CC16 has also been investigated as a biomarker for various diseases, such as asthma [210, 211], ARDS [212], COPD [132, 135], and idiopathic pulmonary fibrosis [213, 214]. A small number of CHE studies have explored the effects of WS and DE on serum CC16 concentration. One CHE-WS study showed serum CC16 concentration was increased by 1.3 µg/L (*p* = 0.004) in 13 healthy participants at 20 h post-exposure to WS for 240 min at a concentration of 240–280 µg/m^3^ PM_1_ [66]. Similarly, another CHE-WS study conducted in 13 healthy participants revealed that exposure to 180 min of WS at 200 µg/m^3^ PM_2.5_ increased CC16 (difference of 1.4 µg/L, *p* = 0.04) at 4 h post-exposure [67]. The same study, however, found no significant effect of WS at a lower concentration of 150 µg/m^3^ PM_2.5_ [67]. A CHE study conducted by Muala and colleagues [69] also demonstrated no correlation between WS (180 min at 300 µg/m^3^ PM_1_) and serum CC16 at 24 and 44 h post-exposure in 14 healthy subjects. Only one CHE study has examined serum CC16 levels with respect to DE in healthy participants; the investigators found no effect of exposure to DE under comparable conditions (180 min at 300 µg/m^3^ PM_1_) to the above WS studies on serum CC16 levels [84].

A small number of CHE studies have also examined the effects of WS and DE on circulating CC16 in atopic participants. In the only relevant CHE-WS study, atopic participants were exposed to 180 min of WS at either 200–400 µg/m^3^ PM, with blood sampled immediately, at 6 h, and at 20 h post-exposure [209]. At both concentrations and at all timepoints, there were no significant effect of WS exposure on serum CC16 [209]. To date, only two CHE-DE studies have examined the influence of DE on serum CC16 in atopic participants, with both studies performed in the presence of allergen co-exposure. In the first study, Biagioni and colleagues [118] exposed 18 atopic participants with or without AHR to DE (120 min at 300 µg/m^3^ PM_2.5_), followed by endobronchial allergen instillation in one lung. At 4, 24, and 48 h post-exposure, there was no effect of DE or allergen co-exposure, in comparison to FA and allergen co-exposure, on circulating CC16 cells [118]. Another CHE-DE study also examined 14 atopic participants with or without AHR, exposing them to inhaled allergens preceded by 120 min of FA, DE (300 µg/m^3^ PM_2.5_), or PDDE, with a control condition of FA exposure and saline inhalation [101]. Allergen exposure was shown to increase serum CC16, with allergen and PDDE co-exposure augmenting this allergen-induced effect at 4 h post-exposure [101]. DE co-exposure did not enhance this effect of allergen, likely reflecting substantially higher NO_2_ concentrations in the PDDE condition [101]. In summary, there is some evidence that implies WS is perhaps a more potent inducer of serum CC16 than DE. Although the scarce quantity of evidence greatly limits the inferences that can be made, the currently available data suggests there may be little effect of either WS or DE on CC16 in atopic populations, with unclear influence of allergens. Like for SP-D and SP-A, as the clinical value of serum CC16 is delineated, CHE studies examining the relationships between WS and DE and this marker may be of greater importance.

### Coagulation

#### DE, but not WS, increases levels of circulating platelets and platelet activation markers

Several CHE studies have examined the effects of DE and WS on markers of thrombosis. In a study conducted by Stockfelt and colleagues [174], 13 healthy participants were exposed to 180 min of WS at either 150 or 200 µg/m^3^ PM_2.5_, with blood sampled at 4, 20, and 44 h after the end of each exposure. Serum platelet counts were decreased at 20 h post-exposure to both WS concentrations and at 4 h post-exposure to WS at 150 µg/m^3^ PM_2.5_ [174]. Soluble P-selectin, a marker of platelet activation, was not significantly affected by WS exposure at any concentration at any timepoint [174]. Two other CHE studies found no effect of WS exposure (120 min at 500 µg/m^3^ PM_2.5_ or 240 min at 250–300 µg/m^3^ PM_1_) on circulating platelet levels in similar sample sizes of 10 or 13 healthy subjects respectively [107, 175]. Similarly, in another CHE-WS study performed in 16 healthy male firefighters, with a shorter duration of 60 min but a much greater PM_1_ concentration of 1100 µg/m^3^, WS had no effect on the serum platelet count at 2–24 h post-exposure [188]. The same study also revealed no effect of WS exposure on markers of platelet activation, such as platelet surface expression of the CD40 ligand and P-selectin [188]. One CHE-WS study conducted in atopic participants similarly demonstrated no effect of WS exposure (180 min at 200 or 400 µg/m^3^ PM_2.5_) on concentrations of soluble P-selectin at timepoints ranging from immediately to 20 h post-exposure [209]. Taken together, the current evidence from CHE studies suggests that WS has little effect on platelet count or platelet activation.

Several CHE-DE studies have demonstrated no effect on serum platelet levels at timepoints ranging from immediately to 24 h after the end of exposure; the majority of these studies were conducted with slightly shorter durations of 60 min (range 30–120 min) compared to the WS studies above, and PM concentrations of 100–350 µg/m^3^ PM [83, 145, 176–178, 181, 183, 189]. Similarly, some CHE-DE studies have also revealed no effect of DE exposures (30–120 min) at concentrations of 200–350 µg/m^3^ PM on markers of platelet activation, such as P-selectin and the CD40 ligand, from immediately to 24 h post-exposure [83, 108, 148, 176, 177, 179]. In contrast, other CHE-DE studies have revealed some evidence of DE-induced increases in circulating platelet counts and platelet activation at similar exposure durations and PM concentrations as most of the CHE-WS studies above. CHE-DE studies conducted in healthy participants exposed to DE for 120 min at PM concentrations ranging from 100 to 300 µg/m^3^ have reported increased levels of circulating platelets at 18–20 h post-exposure [182, 185]. Tornqvist and colleagues [181] demonstrated that DE exposure (60 min at 300 µg/m^3^ PM_10_) increased the serum levels of soluble P-selectin 24 h post-exposure. Similarly, a different CHE-DE study showed that DE exposure (60–120 min at 350 µg/m^3^ PM_10_) increased markers of platelet activation, such as plasma CD40 ligand, platelet–monocyte aggregates, and platelet–neutrophil aggregates [177]. Interestingly, this pro-platelet effect of DE may be enhanced by exercise. In a study conducted by Wauters and colleagues [189], healthy participants were exposed to DE at 300 µg/m^3^ PM_2.5_ while resting for 120 min or while doing intermittent exercise for 60 min. DE exposure had no effect on platelet-monocyte or platelet-neutrophil aggregates during either resting or exercise conditions [189]. However, DE exposure during exercise, but not during resting conditions, was shown to increase the platelet expression of P-selectin and CD63, another marker of platelet activation, in a concentration‒response fashion [189].

Notably, some CHE-DE studies have investigated the effect of DE exposure on circulating platelets in patients with cardiovascular disease (CVD). In a study conducted by Mills and colleagues [194], patients with stable coronary artery disease and a history of previous myocardial infarction were exposed to DE for 60 min at 300 µg/m^3^ PM_10_. At 6 and 24 h post-exposure, there was no significant effect of DE on serum platelet count [194]. A different study by Vieira and colleagues [192] involved patients with heart failure who were exposed to 21 min of DE at 325 µg/m^3^ PM_2.5_ or PDDE at 25 µg/m^3^ PM_2.5_. Immediately post-exposure, there was no effect of DE or PDDE on circulating platelets [192], with the caveat of a much shorter exposure duration compared to that of other CHE studies. To date, no CHE studies have explored the effect of WS on platelet function in patients with clinically significant CVD. Given the known link between platelet dysfunction and the pathogenesis of CVD [215–217], this topic is an important area for future research.

#### Various serum markers suggest a prothrombotic effect of DE but not WS

CHE studies have also investigated the effects of WS and DE on other serum markers of thrombosis. Two CHE-WS studies conducted in healthy never-smokers demonstrated no effect of WS (180–240 min at 150–300 µg/m^3^ PM_2.5_) on serum fibrinogen level at up to 44 h post-exposure [174, 175]. A CHE-DE study also conducted in healthy nonsmokers similarly revealed no effect of DE at comparable duration and PM concentration (180 min at 300 µg/m^3^ PM_1_) on serum fibrinogen immediately or at 20 h post-exposure [84]. In a CHE-DE study including both healthy nonsmokers and healthy smokers, 120 min of exposure to low concentrations of DE (25 µg/m^3^ PM_2.5_) led to a significant increase in fibrinogen (*p* < 0.05) immediately and at 20 h post-exposure according to linear mixed model analyses [186]. The same study also revealed that DE exposure reduced the activity of two endogenous anticoagulants, protein C and protein S [186]. However, CHE-DE studies conducted in healthy participants have not shown a pro-thrombotic effect of DE exposure, ranging from 120 min at 100–200 µg/m^3^ PM_2.5_, on other markers, such as D-dimer and von Willebrand factor (vWF) [183, 218]. CHE-WS studies conducted at slightly longer durations (120–240 min) and higher concentrations (150–500 µg/m^3^ PM_2.5_) have similarly demonstrated no effect of WS on D-dimer or vWF in healthy participants at timepoints ranging from immediately to 44 h post-exposure [107, 174, 175]. Several CHE-WS studies have demonstrated modest effects of WS (180–240 min at 150–300 µg/m^3^ PM_1_) on coagulation factors, with increased levels of factor VIII and factor VII following exposure [174, 175]. Overall, there seems to be limited evidence that DE, but not WS, may induce pro-thrombotic effects on some markers of coagulation.

#### DE may impair endogenous fibrinolytic function, limited evidence for WS

Interestingly, some CHE-DE studies have suggested that DE may impair endogenous fibrinolytic function. In a CHE-DE study conducted in 30 healthy participants, exposure to DE for 60 min at 300 µg/m^3^ PM_10_ attenuated bradykinin-induced release of tissue plasminogen activator (tPA) at 6 h post-exposure (*p* < 0.001) [178]. A different CHE-DE study demonstrated similar DE-mediated attenuation of bradykinin-induced tPA release in participants with coronary artery disease and a history of previous myocardial infarction under comparable exposure conditions and sampling timepoints [194]. However, other CHE studies in healthy participants have shown no effect of DE exposure for the same duration or concentration as Mills et al. [178] on bradykinin-induced tPA release at 6 h [179] or 24 h post-exposure [181]. Similarly, another CHE-DE study conducted in 16 healthy participants demonstrated no effect of DE (120 min at 300 µg/m^3^ PM_10_) on plasma tPA antigen levels or tPA activity at 2, 6, or 24 h post-exposure [190]. The same study also revealed no effect of DE on plasminogen activator inhibitor-1 (PAI-1) [190], further implying that DE has no effect on endogenous fibrinolytic capacity. Other DE studies have also shown no effect of exposure (60–120 min at 100–300 µg/m^3^ PM) on plasma PAI-1 levels in healthy participants at up to 24 h post-exposure [181, 183, 218]. Ghio and colleagues [107] investigated the influence of WS on plasma tPA and PAI-1 in 10 healthy participants and found no effect of an 120-minute WS exposure at a slightly greater PM concentration compared to its CHE-DE counterparts (500 µg/m^3^ PM_2.5_) both immediately and 20 h post-exposure. To date, Ghio et al. [107] is the only CHE-WS study in which the effect on endogenous fibrinolytic function was investigated, underscoring another gap in the literature. While two CHE-DE studies demonstrated evidence of DE-induced impairment of fibrinolytic capacity via assays involving bradykinin-induced tPA release, no CHE-WS studies have used a similar method. It should also be noted that while many CHE-DE studies have not revealed impaired endogenous fibrinolytic function, several of these studies were conducted with lower PM concentrations than those of the two studies that demonstrated a significant response, suggesting that this difference may be concentration dependent.

#### DE increases ex vivo thrombus formation, unclear effect of WS

DE has also been shown to increase ex vivo thrombus formation. In two CHE-DE studies, healthy participants were exposed to DE for 60–120 min at 300–350 µg/m^3^ PM_10_, with blood drawn at 2–6 h post-exposure [177, 179]. Using the Badimon chamber technique, ex vivo thrombus formation was shown to be enhanced by DE exposure [177, 179]. Only one CHE-WS study to date has investigated ex vivo thrombus formation using the Badimon chamber method [188]. In this CHE-WS study, healthy male firefighters were exposed to 60 min of WS at a comparatively high concentration of 1100 µg/m^3^ PM_1_; at 2 h post-exposure, WS did not affect ex vivo thrombus formation [188]. Taken together, these findings, along with evidence from other CHE studies, suggest that DE seems to be more prothrombotic than WS. CHE studies have revealed evidence of DE-induced increases in platelet levels and platelet activation, elevations in some pro-thrombotic markers, impairment of endogenous fibrinolytic capacity, and increased ex vivo thrombus formation. WS has not been shown to have the same pro-thrombotic effect as DE, with many CHE-WS studies using longer durations and/or higher concentrations than their CHE-DE counterparts. However, it should be noted that there are considerably fewer CHE-WS studies on this topic, emphasizing an area for further exploration.

### Cardiovascular

Epidemiological data have demonstrated strong associations between exposure to air pollution and adverse cardiovascular outcomes [219–222]. To substantiate this relationship and add biological plausibility, CHE studies have sought to elucidate the mechanisms governing these relationships using WS and DE as models for common types of air pollution.

#### Exposure to DE increases arterial stiffness, limited evidence for WS

Epidemiological studies have demonstrated positive correlations between arterial stiffness and CVD incidence [223–225]. As such, several CHE studies have investigated the effect of DE and WS on arterial stiffness. Lundback and colleagues [226] exposed healthy participants to 60 min of DE at 350 µg/m^3^ PM_10_. They demonstrated increased arterial stiffness immediately after the end of DE exposure through 20 min post-exposure, with subsequent return to baseline levels at 30 min post-exposure [226]. A different CHE-DE study conducted in healthy smokers and nonsmokers revealed that low-concentration DE exposure (120 min at 25 µg/m^3^ PM_2.5_) increased arterial stiffness immediately and at 24 h post-exposure [186]. Similarly, in a study conducted by Langrish and colleagues [145], arterial stiffness in response to infusion of a nitric oxide synthase (NOS) inhibitor was enhanced by exposure to DE at 300 µg/m^3^ PM_10_ compared to FA. However, one CHE-DE study demonstrated no effect of DE (180 min at 300 µg/m^3^ PM_1_) on arterial stiffness at 1.5 h post-exposure [227]. In a CHE-WS study conducted in 14 healthy subjects under comparable exposure conditions (180 min at 300 µg/m^3^ PM_1_), arterial stiffness increased immediately after WS exposure [228]. Other CHE-WS studies have not shown similar WS-induced increases in arterial stiffness using similar sample sizes [154, 188], although one of these studies was conducted with a shorter duration of 45 min and a slightly lower concentration of 250 µg/m^3^ PM_2.5_ in comparison to their DE counterparts [154]. The other CHE-WS study showing no effect was also conducted with a shorter duration of 60 min but a much greater concentration of 1100 µg/m^3^ PM_1_ [188], suggesting that DE impairs arterial stiffness to a greater extent than WS does. Notably, one CHE-DE study investigated the relationship between DE and arterial stiffness in participants with heart failure and revealed that 21 min of exposure to DE at 300 µg/m^3^ PM_2.5_ decreased arterial stiffness [192]. Given the known association between arterial stiffness and CVD incidence, CHE studies could help delineate the interplay between air pollution, vascular function, and CVD in participants with preexisting CVD.

#### DE impairs endothelium-dependent and endothelium-independent vasodilation, unclear effect of WS

Several CHE studies have investigated the effects of DE and WS exposure on vasodilatory capacity as a corollary of vasomotor function. In several CHE-DE studies, conducted in healthy participants exposed to DE for 60–120 min at concentrations ranging from 250 to 300 µg/m^3^ PM, DE attenuated vasodilation in response to the endothelium-dependent vasodilators bradykinin [176, 178, 179, 190] and acetylcholine [176, 178–181, 190] at up to 8 h post-exposure. In the only WS-equivalent CHE study, 16 healthy subjects exposed to a similar duration (60 min) but a considerably greater concentration of WS at 1100 µg/m^3^ PM_1_ did not demonstrate modification by WS of acetylcholine-induced vasodilation [188]. Interestingly, in this study, WS enhanced bradykinin-induced vasodilation (*p* = 0.003) at 30 min to 1.5 h post-exposure [188]. Hunter and colleagues [188] also examined the effect of WS on vasodilation mediated by the endothelium-independent vasodilators sodium nitroprusside and verapamil, ultimately reporting WS exposure had no effect on vasodilation instigated by these mediators. However, CHE-DE studies have demonstrated that DE at exposure durations of 60–120 min and concentrations of 250–300 µg/m^3^ PM_10_ impaired the vasodilatory response to both sodium nitroprusside [176, 178, 190] and verapamil [176, 179], at up to 8 h after the end of exposure. The effect of DE on endothelium-independent vasodilation may not be as robust as its effect on endothelium-dependent vasodilation, as some CHE-DE studies have demonstrated no modification by DE of the vasomotor response to sodium nitroprusside [179–181] or verapamil [178, 181, 190] in healthy participants exposed to DE for similar duration and at similar PM concentrations. One notable CHE-DE study recruited participants with a history of stable coronary artery disease and previous myocardial infarction, exposing them to DE for 60 min at 300 µg/m^3^ PM_10_ [194]. The investigators found no effect of DE on the blood flow response to acetylcholine, bradykinin, or sodium nitroprusside at 6 to 8 h post-exposure [194], suggesting that there is a more complex interaction between DE, vasodilatory capacity, and established CVD. Taken together, the evidence from CHE-DE studies suggests that DE impairs both endothelium-dependent and endothelium-independent vasodilation, whereas only one CHE study has examined the effects of WS and did not find deleterious effects. Given that impaired vasodilation may be a possible mechanism by which exposure to air pollution negatively impacts cardiovascular health, and some suggestion that DE is more compromising of cardiovascular health than is WS [229–232], this underscores an important area for future research.

#### Acute exposure to DE may increase systolic blood pressure, limited studies of WS

A small number of CHE-DE studies have demonstrated increases in systolic blood pressure induced by acute DE exposure. Rankin and colleagues [233] exposed 16 healthy participants to 40 min of DE at 300 µg/m^3^ PM_10_ and found a 5.1 mmHg increase in systolic blood pressure (SBP) immediately post-exposure. A different CHE-DE study also conducted in 16 healthy participants similarly revealed that DE (120 min at 300 µg/m^3^ PM_10_) significantly increased SBP compared to FA (145 ± 4 vs. 133 ± 3 mmHg, *p* < 0.05) at 6 h after the end of exposure [190]. However, many other CHE-DE studies have demonstrated no effect of DE (ranging from 30 to 120 min at 25–350 µg/m^3^ PM) on SBP or diastolic blood pressure (DBP) at timepoints spanning during exposure to 24 h post-exposure [71, 145, 176, 181, 186, 226, 234–236]. One CHE-DE study conducted in 12 healthy participants reported a 3 mmHg decrease (*p* < 0.05) in SBP with exposure to 120 min of DE at 300 µg/m^3^ PM_2.5_ but no effect of DE on DBP [180]. Another CHE study demonstrated a positive effect of 120 min of DE exposure at 300 µg/m^3^ PM on DBP (5 mmHg increase, *p* = 0.04) at 90 min post-exposure compared to pre-exposure, with no effect of DE on SBP [182].

Fewer CHE studies have examined the effects of WS on blood pressure. One CHE-WS study, using a comparable exposure duration of 45 min and a concentration of 250 µg/m^3^ PM_2.5_, as did Rankin et al. [233] (40 min at 300 µg/m^3^ PM_10_), found no effect of WS exposure on SBP or DBP immediately or at 90 min post-exposure in 10 healthy participants [154]. In comparison, Rankin and colleagues [233] demonstrated an increase in SBP immediately post-exposure, suggesting a greater hypertensive effect of acute DE exposure. It should be noted, however, that several of the CHE-DE studies described above used longer and/or more concentrated exposures than Williamson-Reisdorph et al. [154] but still demonstrated no effect of DE exposure on blood pressure measurements. Similarly, Unosson and colleagues [228] reported no effect of WS (180 min at 300 µg/m^3^ PM_1_) on SBP or DBP in 14 healthy participants immediately or up to 60 min after the end of each exposure. Notably, Hunter and colleagues [188] exposed 16 healthy male firefighters to WS at a much higher PM concentration than those in any other CHE-DE study (1100 µg/m^3^ PM_1_ for 60 min) and found no effect of WS on SBP or DBP at timepoints ranging from immediately to 24 h post-exposure, further suggesting that WS may not provoke a hypertensive response as robust as DE. In the only CHE-WS study thus far to demonstrate a significant effect of WS and blood pressure, 48 healthy participants were exposed to 120 min of WS at four different concentrations (50, 100, 250, 450 µg/m^3^ PM_2.5_), with blood pressure measured beginning immediately to 24 h post-exposure [237]. Immediately after exposure completion, exposure to the highest concentration (450 µg/m^3^ PM_2.5_) decreased SBP by 2.3 mmHg, with no differences observed for the other concentrations [237]. At 24 h post-exposure, SBP was significantly increased by 2–3 mmHg by all WS concentrations except 250 µg/m^3^ PM_2.5_ [237]. The investigators did not observe any significant effect of WS at any concentration on DBP [237]. While this study suggested that acute WS exposure may increase SBP, the equivocal findings of an increase in SBP of the same magnitude suggest that this signal may be artifactual. Nonetheless, these findings only serve to further underscore the need for further investigation on this topic.

Several CHE-DE studies have examined the effect of DE on blood pressure in participants with metabolic syndrome or overt CVD. Metabolic syndrome in these studies was defined as meeting three out of five of the following criteria: elevated waist circumference, elevated triglycerides, decreased HDL cholesterol, elevated SBP or DBP, and elevated fasting glucose, with specific cutoffs for each criterion [238, 239]. Cosselman and colleagues [239] exposed participants both with and without metabolic syndrome to 120 min of DE at 200 µg/m^3^ PM_2.5_. They demonstrated that SBP increased during DE exposure and at up to 20 h after exposure completion, with peak effects occurring at 30 min (3.8 mmHg increase, *p* = 0.08) and 60 min (5.1 mmHg increase, *p* = 0.02) after exposure initiation [239]. However, they described no effect of DE on DBP [239]. In the other CHE-DE study, participants with and without metabolic syndrome were exposed to DE for 120 min at concentrations of 100–200 µg/m^3^ PM_2.5_ [238]. This study reported no effect of DE on neither SBP nor DBP at 30 min post-exposure [238]. Metabolic syndrome has long been associated with CVD [240, 241], highlighting the importance of delineating the interplay between air pollution and blood pressure, another important cardiovascular risk factor, in this population. Other CHE-DE studies conducted in participants with stable coronary artery disease and previous myocardial infarction [194, 242] or heart failure [192] have demonstrated no effect of DE exposure (21–60 min at 300 µg/m^3^ PM) on blood pressure, suggesting that blood pressure impairment by DE may not be as potent in those with established cardiovascular disease. While the CHE-WS studies involved only healthy participants, it would be of great interest for future research to include participants with metabolic syndrome or preexisting CVD.

#### DE may increase heart rate, limited evidence for WS

Several CHE studies have demonstrated positive correlations between DE exposure and heart rate. Wauters and colleagues [180] exposed healthy participants to 120 min of DE at 300 µg/m^3^ PM_2.5_ while resting. Heart rate was increased during exposure to DE compared to ambient air (69 *±* 1 vs. 73 bpm *±* 1, *p* < 0.001) [180]. Stockfelt and colleagues [174] also demonstrated an increased heart rate (*p* < 0.05) during resting exposure to DE (180 min at 300 µg/m^3^ PM_1_) [174]. In a different CHE-DE study, participants with exercise-induced bronchoconstriction but who were otherwise fit and healthy were exposed to 60 min of DE at 300 µg/m^3^ PM_2.5_ and then cycled for 30 min post-exposure [236]. Increases in heart rate were greater with DE exposure compared to FA at 10 min (mean difference 3 bpm, *p* = 0.03) and 70 min (mean difference 4 bpm, *p* = 0.02) postexercise [236]. Giles and colleagues [243] also conducted a CHE study with healthy male participants, similarly exposing them to DE (60 min at 300 PM_2.5_) followed by a cycling bout. DE exposure resulted in a 6-bpm greater increase in heart rate after exercise than did FA (*p* = 0.02) [243]. Several other CHE-DE studies, however, have shown no difference in heart rate in healthy participants with exposure to DE (40–120 min at 200–350 µg/m^3^ PM), both with and without exercise and at timepoints ranging from the start of exposure to 24 h post-exposure [71, 108, 145, 176, 178, 181, 190, 226, 233, 235, 244].

In contrast, only a few CHE studies have examined the effect of WS exposure on heart rate. In one of these studies, 14 healthy participants underwent intermittent exercise during exposure to WS of a similar duration and PM concentration (180 min of WS at 300 µg/m^3^ PM_1_), compared to the CHE-DE studies that demonstrated the positive findings above [228]. The investigators found that heart rate subsequently increased for 1 h post WS exposure (*p* = 0.008) [228]. Other CHE-WS studies have not shown similar WS-induced elevations in heart rate. Williamson-Reisdorph and colleagues [154] reported that a shorter 45-minute exposure to WS at 250 µg/m^3^ PM_2.5_ did not affect heart rate in 10 healthy participants immediately or 90 min post-exposure. Ghio and colleagues [107] demonstrated in 10 subjects, a 16.8% decrease in the maximal heart rate (*p* = 0.02) immediately after exposure to WS for 120 min at 500 µg/m^3^ PM_2.5_ but no difference in heart rate at 20 h post-exposure. A different CHE-WS study, conducted in atopic participants, revealed no effect of 180-minute WS exposure at 200–400 µg/m^3^ PM on heart rate at 6 to 7 h post-exposure [209]. Overall, while DE seems to induce elevations in heart rate, WS does not seem to have the same effect, although there are considerably fewer CHE studies examining WS than DE. Another notable gap in the WS literature is the lack of studies investigating HR in non-healthy populations. CHE-DE studies involving participants with metabolic syndrome [239, 245], coronary artery disease [194], and heart failure [192] have all demonstrated no significant effects of DE exposure on heart rate. Future studies could explore the effects of WS in these populations as well.

#### DE and WS may impair cardiac autonomic function

Heart rate variability (HRV) is a commonly used measure of cardiac autonomic function that has been investigated in several CHE studies. In a CHE-DE study conducted in healthy smokers and nonsmokers, exposure to 120 min of DE at 25 µg/m^3^ PM_2.5_ reduced a time domain measure of HRV (*p* < 0.05) immediately after and at 24 h post-exposure [186]. A different CHE study conducted in healthy participants demonstrated a significant decrease in a frequency domain measure of HRV (*p* = 0.04) 18 h after exposure to DE at 300 µg/m^3^ PM for 120 min, compared to pre-exposure baseline [182]. However, the investigators found no effect of lower concentrations of DE (100 and 200 µg/m^3^ PM) on HRV, nor of any DE concentration on frequency domain indices [182]. Stockfelt and colleagues [227] conducted a CHE-DE study in which ex-smokers and never-smokers were exposed to 180 min of DE at 300 µg/m^3^ PM_1_. They demonstrated a decrease in HRV in the high-frequency band (*p* = 0.02) but no changes in other frequency domain indices during DE exposure [227]. In a CHE-WS study utilizing similar exposure conditions as Stockfelt et al. [227] and the highest concentration of Tong et al. [182], healthy participants were exposed to WS for 180 min at 300 µg/m^3^ PM_1_ [228]. The time domain and frequency domain indices of HRV were significantly lower (*p* < 0.01) during monitoring for 1 h post-WS exposure [228]. Other CHE-WS studies have not demonstrated significant modification of HRV by WS exposure. Ghio and colleagues [107] utilized a similar duration as Tong et al. [182], but at a higher PM concentration of 500 µg/m^3^ PM_2.5_. Under these conditions, Ghio and colleagues [107] found no effect of WS on time domain indices of HRV, although they did demonstrate borderline significant increases (*p* = 0.07 and *p* = 0.1) in some frequency domain measures. A different study performed in atopic participants also revealed no effect of WS under comparable conditions (180 min, 200–400 µg/m^3^ PM) on either time or frequency indices of HRV [209]. Williamson-Reisdorph and colleagues [154] utilized a short duration of 45 min but a similar concentration of 250 µg/m^3^ PM_2.5_, finding no effect of WS on HRV [154]. Several CHE-DE studies have also demonstrated no effect of DE exposure at 300 µg/m^3^ PM, albeit at relatively short exposure durations of 30–40 min, on the HRV in healthy participants [70, 233]. As discussed above, Tousoulis and colleagues [186] demonstrated DE-induced changes in HRV with a much lower concentration of DE (25 µg/m^3^ PM_2.5_) for 120 min, suggesting that this study may be an outlier. Overall, the evidence from CHE studies suggests that DE exposure affects HRV, in turn indicating that DE may impair cardiac autonomic function. The effect of WS is more uncertain given the paucity of CHE-WS studies, underscoring another area for further investigation.

One CHE-DE study also investigated the impact of DE on HRV in participants with metabolic syndrome. Peretz and colleagues [245] exposed participants with or without metabolic syndrome to 120 min of DE at 100–200 µg/m^3^ PM_2.5_, with HRV indices measured at 1 h after exposure initiation to 20 h after the end of exposure. The investigators observed that exposure to DE at 200 µg/m^3^ PM_2.5_ increased HRV in the high-frequency band (*p* < 0.05), and also decreased low-frequency/high-frequency ratio (*p* < 0.01); however, these changes were not consistent between participants [245]. Moreover, DE exposure had no effect on time domain indices of HRV [245]. Another CHE-DE study recruited both healthy participants and those with coronary artery disease and a history of myocardial infarction, exposing them to 60 min of DE at 300 µg/m^3^ PM_10_ [242]. There was no effect of DE exposure on time domain or frequency domain measures in either group [242]. In a different CHE-DE study, participants with heart failure were exposed to 21 min of DE at 300 µg/m^3^ PM_2.5_, with no effect of DE on HRV in either the time or frequency domain [192]. No CHE studies have examined the effect of WS in similar populations, highlighting an area for future research.

#### DE does not appear to be arrhythmogenic, few studies have examined the effects of WS

One notable study pooled data from several CHE-DE studies [176, 178, 190, 194, 246] involving mostly healthy participants but also those with stable coronary artery disease and previous myocardial infarction to investigate the relationship between DE exposure and arrhythmias [247]. The investigators found no effect of DE exposures ranging from 60 to 120 min at 300–350 µg/m^3^ PM_10_ on the occurrence of arrhythmias [247]. The same study also included data from individuals exposed to WS (60 min at 900 µg/m^3^ PM_1_ or 180 min at 300 µg/m^3^ PM_1_), similarly revealing no effect of WS on the incidence of arrhythmias [247]. A separate CHE-DE study also conducted in participants with coronary artery disease and previous myocardial infarction reported no significant arrhythmic events during DE exposure (60 min at 300 µg/m^3^ PM_10_) [242]. A CHE-DE study revealed that DE exposure (21 min at 300 µg/m^3^ PM_2.5_) did not increase the occurrence of significant arrhythmias in participants with heart failure [192]. Taken together, these results suggest that acute exposure to DE and likely WS are not arrhythmogenic in either healthy individuals or those with preexisting CVD, confirming the safety of CHE experiments. It should be noted, however, that the evidence for WS is weaker, with no similar CHE studies being conducted in those with CVD, underscoring a gap in the literature.

### Oxidative stress

#### DE is more potent than WS at inducing systemic oxidative stress

Observational studies have associated exposure to TRAP [248–250] and biomass smoke [251–254] with oxidative stress. Accordingly, this has been a focus of research for CHE studies, using DE and WS as models for air pollution. In a CHE study by Peters and colleagues [195], 10 healthy participants exposed to 90 min of WS at 250–500 µg/m^3^ PM_2.5_ were found to have increased levels of 8-isoprostane and 3-nitrotyrosine (3-NT), markers of oxidative stress [255], in blood samples collected immediately after exposure completion. However, the investigators found no effect of WS concentration on protein carbonyls; interestingly, there was a significant decrease in lipid hydroperoxide (LOOH) (*p* = 0.04) concentration at 1 h post-exposure [195], both of which are also biomarkers of oxidative stress [256, 257]. According to a pooled analysis of both WS concentrations, WS was also shown to decrease plasma levels of the antioxidant uric acid [195]. In a seemingly contrary fashion, blood antioxidant capacity measured via the Trolox equivalent antioxidant capacity (TEAC) assay increased in response to WS exposure (pooled both concentrations and separate analysis of 500 µg/m^3^ PM_2.5_ only) [195], implying an appropriate response to oxidative challenge. Another CHE-WS study demonstrated increased urinary excretion of 8-iso-prostaglandin _2α_ in 13 healthy participants immediately after WS exposure (240 min at 250–300 µg/m^3^ PM_1_) [175], again suggesting oxidative stress [255]. However, Williamson-Reisdorph and colleagues [154], from a study of 10 healthy participants, reported no effect of WS (45 min at 250 µg/m^3^ PM_2.5_) on the serum 8-isoprostane, LOOH, protein carbonyl, or 3-NT concentration, or on the antioxidant capacity measured by TEAC immediately or at 90 min post-exposure. Similarly, Ferguson and colleagues [208] demonstrated no effect of a 90-minute WS exposure at 250–500 µg/m^3^ PM_2.5_ on plasma H_2_O_2_ in 10 healthy participants. Interestingly, Stockfelt and colleagues [174] demonstrated that urinary 8-iso-prostaglandin F_2α_ in 13 healthy subjects was decreased by 50–80% (p *≤* 0.02) when sampled the morning and second morning after exposure to 180 min of WS at 150–200 µg/m^3^ PM_1_. However, there were no changes in urinary 8-Iso-prostaglandin F_2α_ immediately after WS exposure [174]. One possible explanation for the discordant findings from these CHE-WS studies is that Barregard et al. [175] and Peters et al. [195] used sufficiently long and concentrated exposures to elicit observable effects. Williamson-Reisdorph et al. [154] used a relatively short exposure time of 45 min, while Stockfelt and colleagues [174] used comparatively lower PM concentrations. Ferguson et al. [208] used the same exposure conditions as Peters et al. [195] but examined a different marker, perhaps suggesting that plasma H_2_O_2_ may not be as sensitive an indicator compared to other biomarkers.

CHE studies have demonstrated evidence of a stronger effect of DE on systemic oxidative stress. In a CHE-DE study by Wauters and colleagues [180], healthy participants were exposed to 60 min of DE at a concentration of 300 µg/m^3^ PM_2.5_. Endothelial cells incubated with serum from DE-exposed participants produced more reactive oxygen species (ROS) compared to FA-exposed serum [180]. ROS production also correlated with the total amount of inhaled PM_2.5_ [180]. A different CHE-DE study also exposed healthy participants to DE (120 min at 200 µg/m^3^ PM_2.5_), with or without oral antioxidant supplementation prior to exposure [187]. At 3 hours post-exposure, DE decreased oxidized-to-reduced glutathione ratio (GSH/GSSG) in plasma, consistent with oxidative stress [187]. This effect was not attenuated by antioxidant pre-supplementation [187].

Another player is nitric oxide. While it has antioxidant properties, high levels of nitric oxide can also induce oxidative stress via its ability to form potent oxidants, such as peroxynitrite [258–261]. In a study conducted by Giles and colleagues [234], healthy participants were exposed to 30 min of DE at 300 µg/m^3^ PM_2.5_, and blood was collected for measurement of the end products of nitric oxide, nitrite and nitrate (NOx) [234]. Immediately after and at 1 h post-exposure, DE-exposed participants had elevated plasma NOx [234]. Similar results were observed in separate CHE-DE studies demonstrating elevated plasma NOx after exposure to 120 min of DE at 100 µg/m^3^ PM_1_ [262] and elevated plasma nitrite after exposure to DE at 300 µg/m^3^ PM_10_ [145]. Kipen and colleagues [263] reported decreased white blood cell (WBC) and red blood cell (RBC) proteasome activity in healthy participants exposed to 120 min of DE at 200 µg/m^3^ PM_2.5_, which has been associated with oxidative stress [264–266]. A different CHE-DE study demonstrated increased expression of genes related to the oxidative stress response in peripheral blood mononuclear cells (PBMCs) collected from healthy participants exposed to DE (60 min at 300 µg/m^3^ PM_2.5_) [267].

The oxidative effects of DE have also been implicated in asthmatic participants, with some CHE-DE studies revealing DE-induced changes in DNA methylation [268] and microRNA expression [197, 269] in genes associated with oxidative stress. Notably, these CHE-DE studies have predominantly utilized shorter exposure durations and similar, if not lower, PM concentrations than did Barregard et al. [175] one of two CHE-WS studies that supported a link between WS and oxidative stress. A number of these CHE-DE studies also utilized lower PM concentrations than the upper concentration of 500 µg/m^3^ PM_2.5_ used in Peters et al. [195], although most of the DE studies were slightly longer in duration. As such, the current evidence from CHE studies supports DE as a more potent inducer of oxidative stress than WS. It should be noted, however, that some CHE-DE studies have also demonstrated no effect of DE on various markers of oxidative stress in healthy participants, using exposure durations of either 60–180 min and PM concentrations of 300 µg/m^3^ [181, 270, 271]. Tornqvist and colleagues [181] demonstrated DE increased antioxidant capacity at 24 h post-exposure, suggesting positive adaptation to DE-induced oxidative stress. One notable CHE-DE study that did not demonstrate a link between DE and oxidative stress involved participants with metabolic syndrome who were exposed to 120 min of DE at 200 µg/m^3^ PM_2.5_ [272]. At timepoints ranging from 1 to 20 h post-exposure, urinary 8-hydroxy-2’-deoxyguanosine (8-OHdG) and F2-isoprostanes were unaffected by DE [272]. This result is somewhat unexpected, given the established association between oxidative stress and metabolic syndrome [273–275]; perhaps one explanation for the lack of DE-induced oxidative stress is that this population had adapted to chronic oxidative challenge. Future CHE studies could aim to elucidate the effect of DE and WS on oxidative stress in the context of metabolic syndrome, especially considering the lack of CHE-WS studies in this population to date. Another area of interest for new CHE studies is the exploration of antioxidant pre-supplementation and whether this has the potential to mitigate some of the harmful effects of air pollution. While some CHE-DE studies have observed protective effects of oral antioxidant supplementation [110, 197], no comparable studies have been conducted with WS thus far.

#### WS is not a potent inducer of oxidative stress in the airways, DE may induce a protective antioxidant response

The effect of WS on oxidative stress in the airways has also been examined in CHE experiments. Ferguson and colleagues [208] reported that, compared with FA, WS exposure (for 90 min at 250 or 500 µg/m^3^ PM_2.5_) increased 8-isoprostane in the exhaled breath condensate (EBC) of 10 healthy participants at 1 h post-exposure. The same study also revealed no effect of WS on EBC H_2_O_2_ or MPO [208], other markers of oxidative stress [255]. In a different CHE-WS study, 13 healthy participants exposed to 240 min of WS at 250–300 µg/m^3^ PM_1_ were found to have increased EBC levels of the oxidative stress marker malondialdehyde immediately after and at 20 h post-exposure [66]. However, in a separate publication using data from the same study, EBC 8-isoprostane was not affected by WS [276]. Similarly, other CHE-WS studies have failed to support a connection between WS and airway oxidative stress. Muala and colleagues [69] demonstrated no effect of exposure to 180 min of WS at 300 µg/m^3^ PM_1_ on oxidative stress, as indicated by GSSG and MPO in BAL and BW in 14 healthy participants. In a different CHE-WS study also conducted with 19 healthy participants, exposure to 180 min of WS at 200 µg/m^3^ PM_2.5_ had no effect on markers of oxidative stress in BAL and BW, such as GSSG, urate, and ascorbate, at 24 h post-exposure [68]. Another CHE-WS study demonstrated no effect of 180 min of WS at 150–200 µg/m^3^ PM_2.5_ on EBC malondialdehyde in 13 healthy participants at up to 44 h post-exposure [67]. The same absence of effect was also observed in atopic participants in one CHE-WS study examining EBC 8-isoprostane and exposure to 180 min of WS at 200–400 µg/m^3^ PM_2.5_ [73]. Overall, while some CHE studies have produced evidence suggesting a provocative effect of WS on oxidative stress, results from the same studies, as well as separate CHE experiments, fail to support this hypothesis.

Several CHE-DE studies have also explored the interaction between DE and airway oxidative stress. Pourazar and colleagues [277] examined bronchial biopsies sampled from 15 healthy participants at 6 h after a 60-minute exposure to DE at 300 µg/m^3^ PM_10_. They found that DE exposure induced the activation of redox-sensitive transcription factors, suggesting that DE induces oxidative stress [277]. In a different CHE-DE study, 15 healthy participants were exposed to 60 min of DE at 300 µg/m^3^ PM_10_, and nasal lavage, BAL, and BW samples were collected [271]. While DE exposure did not affect levels of GSH, uric acid, or ascorbic acid levels in BW or BAL, the level of ascorbic acid increased in nasal lavage fluid collected immediately after exposure [271], suggesting the induction of protective antioxidant responses in the nasal cavity. Similarly, a CHE-DE study conducted by Mudway and colleagues [106] in 25 healthy participants demonstrated increased GSH (*p* = 0.004), and trend to increased levels of the glutathione precursor L-cysteine (*p* = 0.06) in BW at 6 h post-exposure. The authors also reported increased amounts of GSH and ascorbic acid in nasal lavage at 6 h post-DE exposure [106]. However, DE exposure had no effect on the levels of GSSG, uric acid, or ascorbic acid in BW or BAL, nor did DE affect uric acid in nasal lavage [106]. Another CHE-DE study demonstrated increased levels of GSH and urate in BAL fluid collected from 15 healthy participants at 18 h post-exposure to DE (120 min at 100 µg/m^3^ PM_10_) [78], further suggesting that exposure to DE likely promotes the protective upregulation or mobilization of antioxidants in the airways. One CHE-WS study demonstrated evidence of a similar antioxidant response, with increased GSH levels in BAL after exposure to WS (180 min at 200 µg/m^3^ PM_2.5_) [68]. Notably, in both these CHE-DE studies and in Sehlstedt et al. [68], while increased levels of antioxidants were observed, there were no increases in markers of oxidative stress, implying that the antioxidant response may have been sufficient to protect the airways from oxidative damage. In comparison to their WS counterparts, these CHE-DE studies tended to use shorter exposure durations and, for some, lower concentrations of PM. This may explain why, unlike in CHE-WS studies, most of the CHE-DE studies evaluated did not find any clear provocative effect of DE on markers of oxidative stress. This may also be due to the different sampling sites and biomarkers used, as Ferguson et al. [208] and Barregard et al. [66]. demonstrated increased EBC levels of 8-isoprostane and malondialdehyde, respectively, whereas CHE-DE studies examined only BAL, BW, and nasal lavage fluid levels for other markers. It should also be noted that there were slightly fewer DE studies than WS studies and that the DE studies were all relatively older. In the non-CHE literature, airway oxidative stress has been correlated with exposure to air pollution [278–283]. Air pollution-related pulmonary oxidative stress has also been implicated in the development of systemic inflammation [283], unsurprisingly given that inhalation is the main route of exposure to such pollution. As such, CHE studies play an important role in providing robust evidence of not only causal links between airway oxidative stress and air pollution but also elucidating the mechanisms behind this interaction. More CHE studies, in both DE and WS, are thus needed to fill this current gap in the literature.

### DNA damage

#### Limited number of CHE studies have explored the effects of DE and WS on DNA damage

Few CHE studies have examined the relationship between exposure to DE or WS and DNA damage. In one CHE-WS study, 13 healthy participants were exposed to 240 min of WS at 250–300 µg/m^3^ PM_2.5_, with blood collected 3 and 20 h post-exposure, and urine collected until the morning after exposure [284]. In PBMCs isolated from whole blood, WS decreased levels of strand breaks and increased mRNA expression of the DNA repair enzyme oxoguanine glycosylase 1 (*hOGG1*) [284], which the authors theorized to perhaps represent enhanced DNA repair. WS exposure had no effect on genotoxicity, as assessed via the use of formamidopyrimidine-DNA-glycosylase (FPG) sites, although the investigators did note a trend toward increased urinary excretion of the DNA damage markers 8-oxo-7,8-dihydro-2′-deoxyguanosine (8-oxodG) and 8-oxo-7,8-dihydroguanine (8-oxoGua) [284]. A different CHE-WS study also did not demonstrate a genotoxic effect of WS (180 min at 200 or 350 µg/m^3^ PM_2.5_) in atopic participants, as assessed by strand breaks, endonuclease III, and FPG-sensitive sites [285]. WS exposure also did not affect the expression of *hOGG1* or heme oxygenase-1 (*HMOX1*) mRNA [285]. HMOX1 is thought to exert various cytoprotective effects, including against oxidative damage [286–288]. Few CHE studies have examined the effects of DE on DNA damage. Hemmingsen and colleagues [289] exposed 18 healthy participants to 180 min of DE at 300 µg/m^3^ PM_2.5_ and subsequently examined PBMCs and urine for evidence of DNA damage. Immediately post-exposure, there was no effect of DE on the gene expression of *HMOX1* or *hOGG1* in PBMCs or on the levels of strand breaks, FPG-sensitive sites, or hOGG1-sensitive sites [289]. Moreover, urinary 8-oxodG was not affected by DE exposure [289]. In another CHE-DE study, healthy participants exposed to 120 min of DE at 200 µg/m^3^ PM_2.5_ did not demonstrate changes in the expression of HMOX1 in PBMCs [187]. One CHE-DE study involved participants with metabolic syndrome and revealed no effect of DE (120 min at 200 µg/m^3^ PM_2.5_) on the urinary excretion of 8-hydroxy-2’-deoxyguanosine (8-OHdG) [272], a marker of oxidative DNA damage [290]. The genotoxic effects of both TRAP [291–295] and biomass smoke [296–299] have been repeatedly demonstrated in observational, in vitro, and animal studies. Interestingly, in in vitro experiments directly comparing particles derived from WS and DE, more DNA damage was associated with WS particles than with DE particles [300, 301]. The paucity of CHE studies represents a crucial gap in the literature, one that, if filled, could provide strong support for a causal link between these pollutant exposures and genotoxicity.

## Discussion

Based on the results of CHE studies, exposure to DE appears to be more provocative than exposure to WS in terms of eliciting dysfunctional outcomes (Table 1). Convincing evidence exists for an airway neutrophilic response to DE but not to WS. Similarly, the results of several CHE studies support a systemic neutrophilic inflammatory effect of DE, while findings for WS are less clear (Table 1). Similarly, DE exposure has been shown to likely be a more potent inducer of thrombosis, oxidative stress, and vascular dysregulation (Table 1). Many of these conclusions are based on the observation that significant effects have been found with DE exposures at shorter durations and/or lower PM concentrations than with WS. However, the exposure conditions used in the CHE-DE studies overall tended to be shorter and/or less concentrated in terms of PM than those used in the CHE-WS studies. As such, this may have introduced a sampling bias of some kind; that is, because there are simply more CHE-DE studies, a signal can still be found despite DE experiments using shorter exposure durations and/or lower PM concentrations. Because there are considerably fewer CHE-WS studies, even if one study does demonstrate a significant response to WS, it is more difficult to definitively conclude that this is a “true” response.

A sizeable portion of the reviewed studies reported conflicting outcomes, an issue highlighted by the scarcity of CHE-DE or CHE-WS studies on certain topics. While we do attempt to explain some of these differences in their relevant sections, there are a myriad of caveats and confounding factors in comparing different CHE studies. In this review, we opted to primarily consider exposure duration and PM concentration when comparing WS and DE. Likely, both of these parameters affect outcomes, but the inter-study medley of combinations makes it difficult to discern whether the effects are more time-dependent or concentration-dependent. Few CHE studies have included multiple PM concentrations while controlling for other variables, including duration, providing insight into possible concentration‒response relationships [302]. Another area of incongruity in the CHE literature is the sampled timepoints, again limiting comparisons that can be made between experiments. It should also be noted that the non-PM fractions of WS and DE presumably influence measured outcomes, as has been suggested in animal studies comparing the two exposures [34, 303] and in some CHE-DE studies involving PDDE [101, 304], although this review opted to not focus on these non-PM portions for brevity. Technical aspects of endpoint sampling likely also limit inter-study comparisons, as it is difficult to precisely standardize sampling techniques, equipment, and reagents between studies and research groups. Another area of inconsistency is exercise, as not all studies required participants to exercise during exposure, and for those that did demand this, exercise protocols varied from study to study. Increased ventilation secondary to exercise augments inhalation of PM [189] and likely of the non-PM fractions of pollutants as well, underscoring the need to consider activity levels in inter-study comparisons.

An additional point of heterogeneity specifically regarding DE studies is the evolution of engine tiers over time, which may restrict comparisons between older and newer CHE-DE studies [72, 305, 306]. As well, several different diesel fuels were used in CHE-DE studies [supplement], likely another cause of inter-study inconsistencies. Analogous factors in CHE-WS studies include the lack of standardization of combustion systems and conditions employed in WS studies, as combustion conditions have been shown to impact resultant emissions [27, 30]. It should also be noted that the wood types used in CHE-WS studies also differed considerably [supplement], leading to another source of variation in smoke composition [27, 307] and limiting comparisons that can be made.

Data from in vitro and in vivo experiments directly comparing TRAP and biomass smoke suggest at least some comparable effects of these pollutants [34, 39, 42, 232, 308–311]. PM source-apportion studies have also revealed some similarities in health effects between traffic or diesel air pollution and biomass-burning sources [312], such as with cardiovascular outcomes [313]. Indeed, many epidemiological studies have demonstrated a comparable range of health consequences, such as cardiovascular disease, respiratory morbidity, and low birthweight, with respect to both TRAP [314–316] and biomass burning [17, 22, 317, 318]. Thus, it may be reasonable to extrapolate, to some extent, evidence from CHE-DE studies to the possible effects of WS exposure. In many health outcomes, CHE-WS studies have demonstrated similar results compared to those of their DE counterparts, although definite conclusions from WS studies are limited by the factors discussed above. Indeed, a fairer comparison of DE and WS would be to compare the two within the same experiment to minimize the influence of confounding variables, but this has not been done in human CHE studies to date. Utilizing the same exposure facility, participants, sample collection protocols, etc., would lend much credence to the comparison of WS and DE. Sources of PM vary substantially from region to region [319, 320], underscoring the importance of scrutinizing the effects of different PM sources as well.

**Table 1:** Health effects of diesel exhaust and wood smoke derived from controlled human exposure studies

System	Response	Diesel exhaust	Wood smoke
Pulmonary	eosinophilic inflammation	↔	↔
	neutrophilic inflammation	↑	?
	lymphocytic inflammation	↔	?
	Other inflammatory markers (ICAM-1, MMP-9, IL-6 etc.)	↔	↔
	Pulmonary function	↔	↔
	Airway hyperresponsiveness	↑	?
Systemic inflammation	Neutrophilia	↑	?
	Lymphocytosis	↔	↔
	Monocytosis	↔	?
	Other inflammatory markers (IL-6, ICAM-1, VCAM-1, TNF-α, CRP, etc.)	↑	?
Coagulation	Thrombosis	↑	↔
	Endogenous fibrinolytic function	↓	?
Cardiovascular	Arterial stiffness	↑	?
	Endothelium dependent and independent vasodilation	↓	?
	Systolic blood pressure	↑	?
	Heart rate	↑	?
	Cardiac autonomic function	↓	↓
	Arrhythmias	↔	?
Oxidative stress	Systemic oxidative stress	↑	↑
DNA damage	Genotoxicity	?	?

CHE: controlled human exposure, DE: diesel exhaust, WS: wood smoke, ↑: increase, ↓: decrease, ↔: no effect,? : limited evidence or unclear effect

## Conclusion

The CHE literature of DE is, in most areas, more mature than that of WS. While evidence from CHE studies suggests that DE is a more potent inducer of various dysfunctional outcomes, there are many caveats in performing inter-study comparisons of the effects of DE versus WS. While data from non-CHE experiments and, to some extent, CHE studies suggest that DE-associated effects may be used to inform inferences about WS, this is not ideal. To date, no CHE studies have performed a head-to-head comparison of DE and WS, a notable gap in the literature that should be addressed in future research.

## Electronic supplementary material

Below is the link to the electronic supplementary material.

Supplementary Material 1

## Data Availability

No datasets were generated or analysed during the current study.

## Abbreviations

3-NT: 3-nitrotyrosine
8-OHdG: 8-hydroxy-2’-deoxyguanosine
8-oxodG: 8-oxo-7,8-dihydro-2′-deoxyguanosine
8-oxoGua: 8-oxo-7,8-dihydroguanine
AHR: Airway hyperresponsiveness
AQHI: Air quality health index
ARDS: Acute respiratory distress syndrome
BAL: Bronchoalveolar lavage
BW: Bronchial wash
CHE: Controlled human exposure
CHE-DE: Controlled human exposure to diesel exhaust
CHE-WS: Controlled human exposure to wood smoke
CC16: Club cell protein 16
COPD: Chronic obstructive pulmonary disease
CVD: Cardiovascular disease
DBP: Diastolic blood pressure
DE: Diesel exhaust
EBC: Exhaled breath condensate
ECP: Eosinophil cationic protein
FA: Filtered air
FeNO: Fractional exhaled nitric oxide
FEV1: Forced expiratory volume in 1 s
FPG: Formamidopyrimidine DNA glycosylase
FVC: Forced vital capacity
GSH: Reduced glutathione
GSSG: Oxidized glutathione
HMOX1: Heme oxygenase-1
hOGG1: Oxoguanine glycosylase 1
HRV: Heart rate variability
ICAM: Intracellular adhesion molecule
IL: Interleukin
LAIV: Live attenuated influenza virus
LOOH: Lipid hydroperoxide
MMP-9: Matrix metalloproteinase-9
MPO: Myeloperoxidase
NOS: Nitric oxide synthase
NOx: Nitrite and nitrate
PAI-1: Plasminogen activator inhibitor-1
PBMC: Peripheral blood mononuclear cell
PDDE: Particle-depleted diesel exhaust
PM: Particulate matter
RBC: Red blood cell
ROS: Reactive oxygen species
SBP: Systolic blood pressure
SP-A: Surfactant protein A
SP-D: Surfactant protein D
TEAC: Trolox equivalent antioxidant capacity assay
Th2: T helper 2
tPA: Tissue plasminogen activator
TNF-a: Tumor necrosis factor-alpha
vWF: von Willebrand factor
WBC: White blood cell
WS: Wood smoke
